# Controlling Fibronectin Fibrillogenesis Using Visible Light

**DOI:** 10.3389/fmolb.2020.00149

**Published:** 2020-07-08

**Authors:** Tetyana Gudzenko, Clemens M. Franz

**Affiliations:** ^1^DFG-Center for Functional Nanostructures, Karlsruhe Institute of Technology, Karlsruhe, Germany; ^2^WPI Nano Life Science Institute, Kanazawa University, Kanazawa, Japan

**Keywords:** fibronectin, fibrillogenesis, AFM, matrix nanomechanics, visible light

## Abstract

We previously developed a surface-assisted assay to image early steps of cell-induced plasma fibronectin (FN) fibrillogenesis by timelapse atomic force microscopy (AFM). Unexpectedly, complementary attempts to visualize FN fibrillogenesis using fluorescently labeled FN (Alexa Fluor 488 or 568) and live-cell light microscopy initially failed consistently. Further analysis revealed that fibrillar remodeling was inhibited efficiently in the focal area illuminated during fluorescence imaging, but progressed normally elsewhere on the substrate, suggesting photo sensitivity of the FN fibrillogenesis process. In agreement, active cell-driven fibrillar extension of FN could be stopped by transient illumination with visible light during AFM timelapse scanning. Phototoxic effects on the cells could be ruled out, because pre-illuminating the FN layer before cell seeding also blocked subsequent fibrillar formation. Varying the illumination wavelength range between 400 and 640 nm revealed strong inhibition across the visible spectrum up to 560 nm, and a decreasing inhibitory effect at longer wavelengths. The photo effect also affected unlabeled FN, but was enhanced by fluorophore labeling of FN. The inhibitory effect could be reduced when reactive oxygen species (ROS) were removed for the cell imaging medium. Based on these findings, FN fibrillogenesis could be imaged successfully using a labeling dye with a long excitation wavelength (Alexa Fluor 633, excitation at 632 nm) and ROS scavengers, such as oxyrase, in the imaging medium. Fibrillar remodeling of exposed cell-free FN layers by AFM scanning required higher scan forces compared to non-exposed FN, consisting with mechanical stiffing of the FN layer after illumination. In agreement with changes in FN mechanics, cells spreading on pre-exposed FN showed reduced migration speeds, altered focal adhesion arrangement, and changes in mechanosensitive signaling pathways, including reduced FAK (Y397) and paxillin (Y118) phosphorylation. Pre-exposure of FN to visible light prior to cell seeding thus provides a useful tool to delineate mechanosensitive signaling pathway related to FN fibrillogenesis. When using FN-coated cell adhesion substrates, care should be taken when comparing experimental results obtained on non-exposed FN layers in cell culture incubators, or during live-cell fluorescence imaging, as FN fibrillogenesis and mechanosensitive cellular signaling pathways may be affected differently.

## Introduction

Fibronectin (FN) is a large dimeric extracellular matrix glycoprotein with a wide range of functions during embryogenesis, tissue homeostasis, and wound healing (Grinnell, 1984; Pankov and Yamada, 2002; Zollinger and Smith, 2017). Fibronectin is also an important matrix scaffold and cell adhesion protein, providing cell anchorage to the extracellular matrix via integrin receptor binding sites (Singh et al., 2010; Schwarzbauer and DeSimone, 2011). There are several closely related isoforms of FN generated by alternative splicing from a single gene (Schwarzbauer et al., 1983). Soluble plasma FN is produced by hepatocytes and released into the blood stream and circulates until it becomes activated at sites of tissue injury (Zardi et al., 1979). There it is incorporated into fibrin clots, providing a scaffold for subsequent cell recruitment and helping to re-establish tissue integrity. In contrast, cellular FN is synthesized by fibroblasts and other cell types and incorporated directly into the surrounding extracellular matrix for cross-linking and stabilization (To and Midwood, 2011), growth factor attachment and providing a scaffold cell adhesion and for guiding cell migration (Boucaut et al., 1990; Erdogan and Webb, 2017; Yang et al., 2017).

Both plasma and cellular FN are initially secreted in a compact, inactive conformation (Hynes, 1985) and do not polymerize or form 3D matrices in absence of cells (Mosher and Johnson, 1983; Mao and Schwarzbauer, 2005). Rather, the biological functions of FN are closely linked to its ability to extend and to form fibrillar assemblies in a cell-mediated process (McKeown-Longo and Mosher, 1983). Actomyosin-dependent forces transmitted by integrin receptors at cell-matrix adhesion sites unfold FN into an extended, active conformation (Mosher, 1993; Dzamba et al., 1994; Halliday and Tomasek, 1995; Wu et al., 1995; Christopher et al., 1997; Sechler et al., 1997). The molecular extension of FN exposes FN–FN binding sites, enabling FN molecules to interact laterally and to assemble into fibrillar complexes (McDonald, 1988; Hocking et al., 2000; Singh et al., 2010). Individual FN fibrils can be bundled by cells and further remodeled into complex, branched fibrillar tissue matrices (Chen et al., 1978; Singer, 1979). The unique capability of FN for extension is founded in its particular molecular architecture. The multi-nodular domain structure of FN featuring arrays of FN type I, II, and III repeats (Hynes, 1985) provides sufficient flexibility for extension (Erickson et al., 1981; Rocco et al., 1987), while an RGD sequence within the FNIII10 repeat serves as a binding site for α5β1 integrin (Wu et al., 1993). The dimeric structure of FN mediated by disulfide bonds at the C-terminus doubles these attachment points and enables cells to create sufficient tension across individual FN dimers for unfolding and extension (Mao and Schwarzbauer, 2005).

Important insight into the multistep process of cell-driven FN fibrillogenesis has been obtained by fluorescence microscopy. For instance, fixing cells incubated on fluorescently labeled FN after different time points can generate snapshot images of different stages of FN fibrillogenesis (Avnur and Geiger, 1981; Pankov et al., 2000; Pankov and Momchilova, 2009). Moreover, these studies have shown that α5β1 integrin receptors clustered in cellular adhesions translocating from the cell periphery toward the cell center stretch FN molecules against the substrate and drive fibril formation (Pankov et al., 2000; Ohashi et al., 2002). However, FN fibrillogenesis is a highly dynamic process and imaging fixed samples poses the potential risk of missing intermediate stages. Transfection with a FN-YFP expression construct permitted real-time observation of the rearrangement of larger FN fibrils forming after several hours to days in cell culture and provided valuable information about the rate and extent of fibrillar extension (Ohashi et al., 2002; De Jong et al., 2006), but this technique has not been used to study early steps of FN fibrillogenesis.

Förster resonance energy transfer (FRET)-experiments have provided important insight into conformational changes occurring to the FN molecule during cell-induced FN fibrillogenesis (Smith et al., 2007). However, studies using conventional light microscopy techniques are unsuitable to resolve the earliest steps of fibrillogenesis occurring at the nanoscale. Atomic force microscopy (AFM)-based imaging has been used to visualize molecular-scale structural changes of FN fibrils extended through cellular (Gudzenko and Franz, 2015) or external forces (Szymanski et al., 2017). Nevertheless, AFM live-cell imaging still suffers from limited scan speeds and therefore cannot adequately time-resolve the earliest steps of FN fibrillogenesis, which occur on the scale of seconds to minutes. Faster, optical imaging techniques, ideally incorporating optical superresolution (Früh et al., 2015), are therefore highly desirable for visualizing the earliest steps of FN fibrillogenesis by living cells in real-time. Surprisingly, however, reports using optical microscopy to visualize early steps of FN fibrillogenesis directly in living cells are sparse.

## Materials and Methods

### Cell Culture and Transfection

Rat embryonic fibroblasts (REF), mouse embryonic fibroblasts (MEF), and human foreskin fibroblasts (HFF) were cultured in Dulbecco’s modified Eagle’s medium (DMEM) containing 10% fetal bovine serum, 100 IU/ml penicillin, and 100 μg/ml streptomycin at 37°C and 5% CO_2_. Cells were passaged every 2–3 days or before reaching confluency. Mouse embryonic fibroblasts were transfected with a vinculin-GFP expression construct by electroporation as described previously (Fichtner et al., 2014).

### Fibronectin Labeling and Surface Coating

Lyophilized human plasma FN (Roche, Grenzach-Wyhlen, Germany) was resuspended in sterile water and stored at −80°C. Fibronectin was labeled by conjugating Alexa Fluor 488, Alexa Fluor 568, or Alexa Fluor 633 succinimidyl (NHS) ester dyes to primary amines of FN using the Alexa Fluor Protein Labeling Kit^1^. Before labeling FN (2 mg/ml) was dialyzed against PBS in a 2K dialysis cassette overnight at 4°C and then incubated with the Alexa Fluor dye at room temperature in the dark for 1 h. Unbound dye was removed from the conjugate by gel filtration using a Sephadex G-10 column. Final elution was performed using PBS. The degree of labeling was determined by photospectrometry according to the manufacturer’s instructions (5–8 moles of dye per mole Alexa Fluor 488, 2–6 moles of dye per mole Alexa Fluor 468, and 1–3 moles of dye per mole Alexa Fluor 633). Fibronectin-Alexa Fluor conjugates were stored at a concentration of 1 mg/ml in PBS at −80°C. For surface coating, unlabeled FN or fluorescently labeled FN conjugates were diluted in PBS to a final concentration of 50 μg/ml and centrifuged at 15,700 rcf for 5 min to separate protein aggregates from the solution. Afterward, glass bottom cell culture dishes (Fluorodish, FD35)^2^ or freshly cleaved mica disks were coated with the FN solution at room temperature for 1 h in the dark and finally rinsed with PBS to remove unbound proteins.

### Exposure of FN to Visible Light

Fibronectin-coated substrates (FD-35 Fluorodish) were maintained in PBS during exposure at different wavelengths (400, 440, 480, 520, 560, 600 and 640 nm) using a monochromator light source (Polychrome 5000)^3^ equipped with a 150 W xenon lamp. The monochromator permits adjusting the wavelength in ∼15 nm units (half-power bandwidth). The light was focused on the FN-coated surface through a LD Plan-Neofluar 63×/0.75 objective^4^. The position of the probe was changed after each illumination step so that different areas on the same substrate could be exposed with different wavelengths. To ensure that all sample areas received an equal number of photons regardless of wavelength, the incident light power at each wavelength was first measured with a power meter and the exposure time was then adjusted accordingly. The energy of a photon *E*_λ_ equals

$$E_{\lambda }=\frac{h\cdot c}{\lambda },$$

where *h* is the Planck’s constant, *c* is the velocity of light and λ is the wavelength. The number of incident photons *N*_λ_ equals

$$N_{\lambda }=\frac{P_{\lambda }\cdot t_{\lambda }}{E_{\lambda }},$$

where *t*_λ_ is the exposure time and *P*_λ_ is the incident light power as a function of wavelength. Different exposure times were normalized to the reference condition at 520 nm with *P*_520_ = 45 μW (per mm^2^) and *t*_520_ = 5 min. Thus, the exposure time as a function of the wavelength is

$$t_{\lambda }=\frac{P_{520}\cdot 520\,\text{nm}\cdot t_{520}}{P_{\lambda }\cdot \lambda }.$$

For time-dependent exposure, FN-coated FD35 glass substrates submerged in PBS were exposed using irradiation times ranging from 1 to 300 s using a Zeiss Axio Observer inverted optical microscope and an X-Cite 120 xenon lamp^5^. Via an optical band pass filter, illumination wavelengths were restricted to a range between 480 and 490 nm, and the beam was focused on the sample with a 63× objective (LD Plan-Neofluar 63x/0.75).

### FN Exposure Through Photo Masks

Photo masks were produced by printing inverted patterns on transparent film using a commercial laser printer. The film was then attached to the underside of an FD35 glass bottom dish coated with FN-AF488. A substrate area of ∼1.5 cm^2^ was exposed through the mask for 10 min at a power of 76 μW/mm^2^ using unfocused light, an Axio Observer inverted optical microscope and an X-Cite 120 Xenon lamp (120 W). Alternatively, the FD35 dish carrying the photo mask was exposed at 365 nm on a Bright Light table at a power of 100 μW/mm^2^ for 5 min. The exposed substrates were then washed once in PBS and MEF cells were seeded and cultured in serum-free DMEM for 16 h and fixed. Multiple fluorescence images covering the entire exposed substrate were obtained using a 20× objective and assembled into a single image collage using Adobe Photoshop.

### Immunofluorescence Staining, Epifluorescence and Total Internal Reflection Fluorescence Imaging

For immunostaining cells growing on FN substrates were fixed for 10 min with 4% PFA, permeabilized with PBS containing 0.2% Triton X-100 for 5 min and incubated with primary antibody to paxillin (mouse monoclonal)^6^, vinculin (V9131 mouse monoclonal)^7^, or FN (F-3648 rabbit polyclonal anti-fibronectin antibody)^7^ at room temperature for 1 h. After three wash steps with PBS containing 0.5% BSA, samples were incubated with the corresponding secondary antibodies for 1 h at room temperature and finally washed with PBS. Cell nuclei were stained with 4,6-diamidino-2-phenylindole (DAPI). Epifluorescence images were collected using an iMIC microscope (FEI Life Sciences, Munich, Germany) and APON 60xOTIRF or UPLSAPO 40x2 objectives^8^. Fibronectin-AF633 substrates were imaged using an Axio Imager 2 microscope, a Colibri LED illumination system and a 40× Fluar oil immersion objective^4^. For Total internal reflection fluorescence (TIRF) imaging, cells were seeded into FN-coated FD35 glass bottom dishes in serum-free DMEM containing 20 mM HEPES pH 7.6, 1% penicillin/streptomycin and 20 mM HEPES pH 7.6 and imaged immediately for live cell experiments or after incubation for 16–24 h and chemical fixation. Live-cell TIRF imaging on FN-AF488 or FN-AF568 was performed on an iMIC microscope with an APON 60x OTIRF objective (Olympus) using a 491 nm (100 mW) or 561 nm (75 mW) diode-pumped solid state laser. All live-cell imaging was performed at 37°C. The generated images were processed with the ImageJ software. In experiments assessing the role of ROS, serum-free DMEM was supplemented with a cocktail of free-radical scavengers (10 mM DL-lactate, 5 mM L-ascorbic acid, 0.3–0.6 U/ml Oxyrase), as well as 1 mM MnCl_2_ to activate integrins.

### Western Blotting

Cells were seeded in glass-bottom tissue culture dishes (WPI DF35 Fluorodish or Matsunami) at a density of 500 cells/mm^2^ and cultured for 16 h. Afterward, cell lysates were prepared in cell lysis buffer (1% Triton X-100, 150 Mm NaCl, 10 mM Tris buffer (pH 7.5), 1mM EDTA (pH 8.0) and 1 tablet/ml proteinase inhibitor cocktail), separated by SDS-PAGE and transferred onto PVDF membranes. Membranes were then blocked with 5% milk in TBS-T (TBS buffer containing 0.1% Tween) for 1 h at room temperature, incubated with primary antibodies (rabbit monoclonal anti-phospho-FAK (pY397, Invitrogen), mouse monoclonal anti-FAK (BD Transduction Labs), rabbit polyclonal anti-phospho paxillin (pY118, Cell Signaling Technologies), mouse monoclonal paxillin (BD Transduction Labs), or β-actin (mouse monoclonal, Sigma) and then with corresponding HRP-conjugated secondary antibodies. Blots were developed using Western Lightning Plus-ECL substrate.

### Atomic Force Microscopy Imaging

Atomic force microscopy imaging was performed using a JPK NanoWizard II scanner mounted on top of an AxioObserver inverted optical microscope. For AFM time-lapse imaging of cell-mediated FN fibrillogenesis, a mica disk (Indiamart, Noida, India) glued into a cell culture dish was freshly cleaved and coated with unlabeled FN at a concentration of 50 μg/ml at room temperature in the dark for 1 h. The substrate was subsequently washed once with serum-free DMEM containing 20 mM HEPES pH 7.6, and 1% penicillin/streptomycin. Rat embryonic fibroblasts cells were then seeded in the same medium and immediately scanned at 37°C in contact mode using gold-coated silicon nitride V-shaped cantilevers (MLCT-C) with a nominal spring constant of 0.06 N/m and line scan rates between 0.3 and 2.5 Hz. Atomic force microscopy images were processed using the JPK image processing software. For AFM rearrangement experiments, FN-coated mica disks (50 μg/ml in PBS) were washed with PBS. For thermal denaturation, the substrates were incubated for 30 min at 60°C in a heated incubator prior to scanning. For chemical fixation substrates were then incubated with 1% glutaraldehyde/PBS for 30 min and then washed with PBS, 0.1% BH4/PBS for 5 min to quench free aldehyde groups, and finally washed again with PBS. In exposure experiments the FN substrates were illuminated on an Axio Observer inverted light microscopy with a X-Cite 120 lamp at 9 mW/cm^2^ for 10 min and then rinsed with PBS. Atomic force microscopy rearrangement experiments were performed in PBS at room temperature in contact mode using MLCT-C cantilever with a nominal spring constant of 0.06 N/m. To ensure reproducibility in force application, the sensitivity and spring constant of cantilevers were calibrated before each experiment. First, 15 × 15 μm^2^ overview scans of FN-coated areas were collected using a minimal scan force of ∼0.1 nN to verify homogenous FN distribution within the scan area. Using a low scan force prevented potential rearranging the FN layer through lateral scanning forces exerted by the AFM tip. Afterward, 3 × 3 μm^2^ sections within the overview area were scanned applying contact forces between 0.1 and 6 nN. After manipulation, the same overview area of 15 × 15 μm^2^ was re-imaged at minimal contact force (<0.1 nN) to visualize local FN rearrangement during the previous high force scanning steps.

### Image Analysis

To quantify cell migration, cells were seeded on substrates immediately before imaging and observed with the iMIC microscope and an UPLAPO 10x2 lens. Images were collected every 30 s using bright field illumination for a total duration of 6 h and cell migration was analyzed using the Manual Tracking plugin in ImageJ. To determine cell shape and cell area, cells were imaged by phase contrast using a 20× Plan-Apochromat objective. Cell borders were outlined and cell shape and area parameters were analyzed in ImageJ. To quantitate focal adhesion size and arrangement, cells were stained for vinculin as a marker protein and fluorescence and phase contrast images of the cells were then collected using a 40× Plan-Apochromat lens. From the fluorescence images, focal adhesions were separated from background signals by applying a brightness threshold in ImageJ. Using the Analyze Particle Plugin, focal adhesion area, length, width and roundness (width over length) were extracted. Cell outlines were superimposed the fluorescence images of vinculin to calculate the distance of each focal adhesion to the cell border. The obtained values were plotted as histograms or bar graphs plots using OriginPro 8.6G. Statistically significant differences between conditions (*p* < 0.05, 0.01 and 0.001) were denoted as one, two or three asterisks.

## Results

### Visualizing Cell-Induced FN Remodeling at Focal Adhesions

**FIGURE 1:**
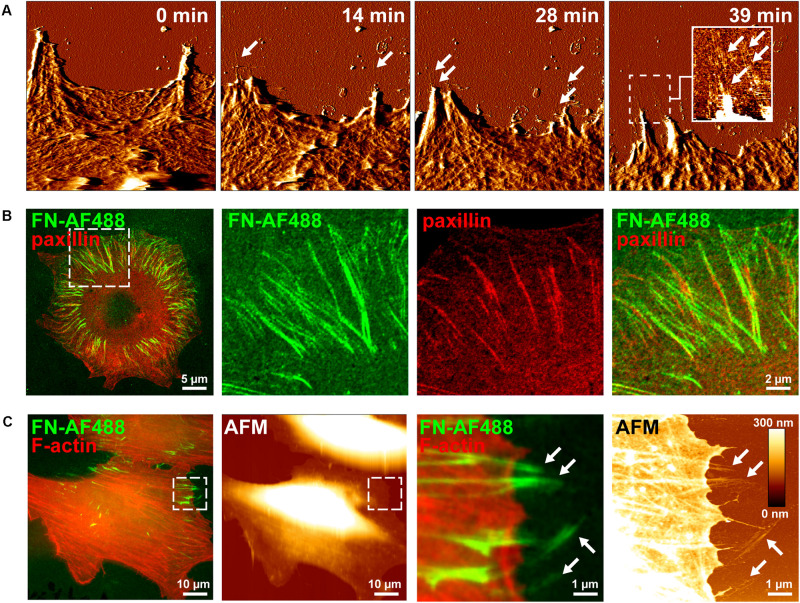
Visusalizing cell-induced FN fibrillogenesis by AFM and fluorescence microscopy. **(A)** Individual MEF cells were seeded on a homogenous FN layer adsorbed onto mica disks for 1 h and then imaged by continuous AFM contact mode scanning. Representative image frames (error channel) extracted from the timelapse series (see Supplementary Movie 1) show the gradual creation of FN nanofibrils at sites of membrane retraction (arrows). An insert in the left panel shows a magnified view (height image) of the created FN nanofibrils of the area denoted by the dashed box. Size of the AFM timelapse images 10 × 10 μm^2^, full range of the height scale (insert) is 15 nm. **(B)** Mouse embryonic fibroblasts (MEFs) expressing incubated on Alexa Fluor 488-labeled fibronectin (FN-AF488) for 1 h, fixed and immunostained for paxillin. Merged (left panel) and higher magnification single and merged channel images (center and right panels) corresponding to the area denoted by the dashed box. **(C)** Complementary AFM height and TIRF microscopy images of MEFs incubated on FN-AF488 for 1h after fixation and staining for F-actin. The areas denoted by the dashed boxes (left panels) shown at high magnification (right panels).

Cell-induced FN fibrillogenesis has been previously studied by seeding fibroblasts or other cell types onto glass substrates homogenously coated with a homogenous FN layer, which cells then remodel into fibrillar structures over the course of several hours (Avnur and Geiger, 1981). In a previous study we had employed a similar mica surface-assisted assay and live-cell AFM imaging to reveal a step-wise extension mechanism of nascent FN fibrils during membrane retraction of fibroblast cells (Gudzenko and Franz, 2015). Atomic force microscopy is a surface scanning method and therefore cannot image nascent FN fibrils forming in central areas of the basal cell side. However, in REF cells membrane retraction coincides with a simultaneous translocation of peripheral focal adhesions in the same direction. These translocating focal adhesions typically locate near the very cell edge, as they are the last cellular structures providing significant resistance to membrane retraction. As a result, FN fibrils forming at focal adhesion right next to the cell edge become immediately exposed during cell membrane retraction and can be readily imaged by AFM (Figure 1A and Supplementary Movie 1). However, while generating high-resolution images of nascent FN nanofibrils, conventional live-cell AFM usually permits only comparatively low frame rates (typically ∼ 1 to 5 min per frame) and therefore cannot adequately time-resolve the earliest steps of FN fibrillogenesis, which likely occur on the second to minute scale. Moreover, AFM images contain no direct information regarding the molecular identity of the imaged structures. This can complicate the identification of the FN nanofibrils in the AFM scans, although FN nanofibrils (typical height ∼10 nm) can be unequivocally distinguished from cellular structures (>60 nm) based on their different height in AFM images (Gudzenko and Franz, 2015).

In contrast to AFM, fluorescence microscopy permits observing labeled proteins at high frame rates, presenting itself as a potential technique to observe the dynamic rearrangement of FN. In an attempt to visualize different stages of cell-induced FN fibrillogenesis at higher temporal resolution by fluorescence microscopy, we labeled FN with the Alexa Fluor 488 fluorescent dye (FN-AF488) prior to adsorption to glass substrates. To confirm that fluorescent labeling of FN did not impede cell-dependent fibrillogenesis, MEFs were incubated on FN-AF488 substrates in a cell culture incubator for 4 h and fixed. Total internal reflection fluorescence microscopy images demonstrated that cells had remodeled the initially homogenous FN-AF488 substrate into radial fibrillar arrays (Figure 1B). Immunostaining for paxillin and TIRF imaging confirmed that FN fibrillogenesis occurred at focal adhesion sites (Figure 1B), consistent with the requirement of cellular traction forces for this process. Combined fluorescence microscopy and AFM scanning of fixed cells on AF488-FN furthermore confirmed that FN fibrils often formed at the cell periphery, where they extend beyond the cell margin where they become accessible to the AFM tip (Figure 1C). When cells were seeded at higher density (>200 cells/mm^2^), cells cooperatively remodeled the underlying FN layer into complex interconnected fibrillar arrays (Supplementary Figure 1), yielding a fully restructured FN layer after 16 h (Supplementary Figure 2).

### Inhibition of FN Fibrillogenesis During Fluorescence Live Cell Imaging

**FIGURE 2:**
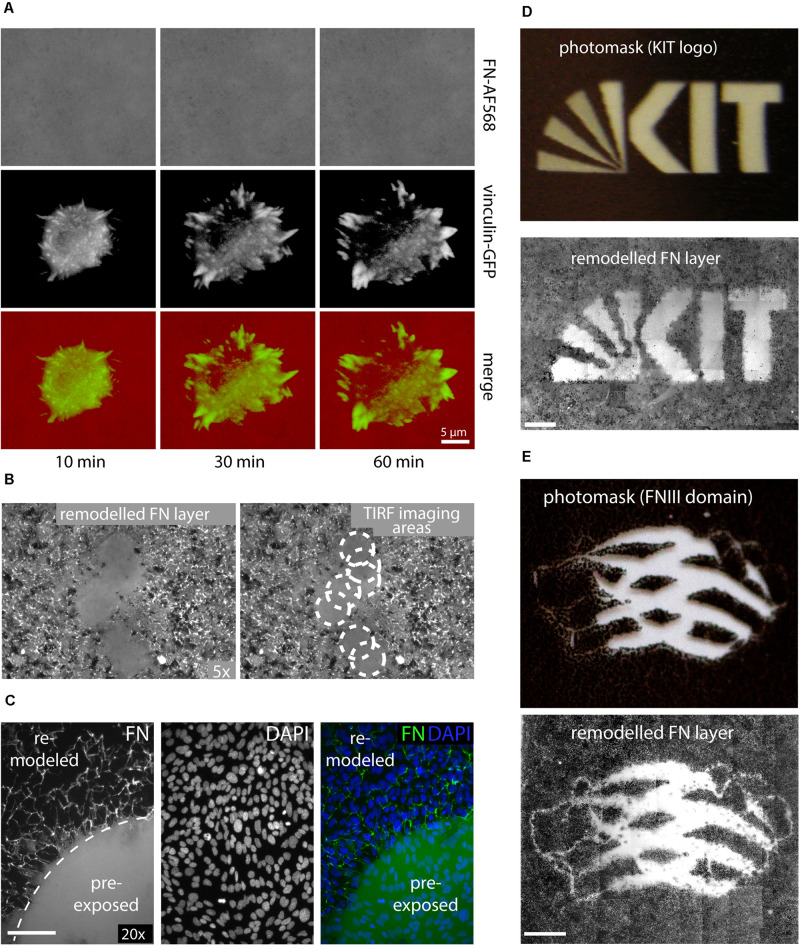
**(A)** Live-cell TIRF imaging of a representative MEF cell expressing vinculin-EGFP seeded on Alexa Fluor 568-labeled FN (FN-AF568). Still images extracted from a time-lapse series (Supplementary Movie 2) of FN-AF568 (top row), vinculin-EGFP (middle row), and overlay (bottom row) 10, 30, and 60 min after cell seeding. Despite normal cell spreading behavior and focal adhesion formation, the FN layer is never remodeled. **(B)** Overview image (5× lens) of the same FN substrate imaged in panel **(A)** with by TIRF (63× lens). White circles indicate areas of non-remodeled FN, corresponding to different positions of the TIRF imaging plane. **(C)** Pre-illumination of a circular area on a FN-AF488 layer before cell seeding using a 491 nm laser, a laser power of 10 mW and an exposure time of 10 min. Subsequently, a dense MEF monolayer was seeded and cultivated on the FN layer in the dark for 16 h and finally fixed. DAPI staining demonstrates a homogeneous cell distribution across the substrate, but FN fibrillogenesis is completely inhibited in the pre-exposed area. **(D)** Exposing FN-AF488 through photomask masks depicting the KIT logo or panel **(E)** a model of the FN type III domain at 0.1 mW for 5 min. After MEF incubation (16 h) and fixation, approximately 50 overlapping areas on each substrate were imaged using a 10× objective and the collected fluorescence images were aligned using Photoshop. Scale bar 200 μm.

In contrast to the above experiments analyzing FN fibrillogenesis of cell samples fixed after removal from an incubator, attempts to visualize dynamic FN remodeling by life-cell TIRF microscopy unexpectedly failed consistently. In part of these experiments, MEFs were transfected with a vinculin-EGFP expression construct for simultaneous imaging of dynamic focal adhesion rearrangement prior to seeding on AFN-AF568. Imaging of single cells revealed normal spreading behavior and dynamic focal adhesion assembly and disassembly (Figure 2A and Supplementary Movie 2), consistent with the formation of functional cell adhesion contacts. Nevertheless, the FN layer remained homogenous and completely free of FN fibrils throughout the duration of the timelapse experiment (1 h). The complete absence of fibrillar structures in light microscopy timelapse experiments was in stark contrast to the results obtained when cells were grown in a cell culture incubator, which revealed extensive fibrillar remodeling at corresponding incubation times (60 min, compare Figures 1C, 2). However, lower magnification overview epi-fluorescence images of the FN substrate collected with a 5× objective after completion of TIRF imaging (63× objective) showed that fibril-free areas were restricted to discrete circular regions, while fibrillogenesis had occurred normally elsewhere on the substrate (Figure 2B). The circular areas of non-remodeled FN corresponded precisely to different substrate regions that had been illuminated during the preceding live cell TIRF imaging (Figure 2A). Apparently, local sample illumination by TIRF had blocked FN fibrillogenesis without affecting focal adhesion dynamics or cell viability. To exclude that light-induced changes in cell behavior during live-cell imaging caused the suppression of FN fibrillogenesis in the imaging plane, we pre-illuminated a single circular area (corresponding to the field of view of the 63× TIRF oil immersion lens) on a cell-free FN substrate for 10 min (491 nm laser, 10 mW laser power), and subsequently cultivated a dense monolayer of cells on this substrate in the dark for 16 h. DAPI staining to visualize cell nuclei in fixed cells showed that cells had grown to comparable densities in the pre-illuminated and the non-illuminated control region (Figure 2C). However, similar to the live-cell TIRF experiments, FN fibrillogenesis occurred exclusively in the non-pre-illuminated control area. The border between the illuminated and non-illuminated areas revealed an abrupt transition from a fibril-free to a fibril-rich area (Figure 2C), demonstrating that FN fibrillogenesis can be controlled with high spatial precision by selective illumination of the FN substrate. While the original fluorescence signal of the labeled FN substrate was preserved within the pre-illuminated area, cell-induced fibrillar bundling of FN caused an apparent decrease in average fluorescence intensity in the remodeled areas (Supplementary Figure 3). The inhibitory effect could be observed when using different FN concentrations for surface coating (Supplementary Figure 4) or different types of glass substrates (Supplementary Figure 5), and pre-exposed FN layers resisted cell-dependent fibrillar remodeling for up to at least 72 h of cell culture (Supplementary Figure 6).

To further demonstrate the possibility to pattern FN fibrillogenesis through selective illumination, photo masks were produced by printing different millimeter-sized binary patterns (molecular model of a single FN type III domain, KIT Logo) onto transparent film using a commercial laser printer (Figures 2D,E and Supplementary Figure 7). Homogeneous FN-AF488 substrates were then pre-exposed through these masks at a power density of 0.1 mW/cm^2^ for 5 min. Afterward, MEF cells were seeded on the substrates, incubated for 16 h and fixed. Subsequent fluorescence imaging of the FN layer revealed patterns that correlated well with the respective macroscopic photomasks (Figures 2D,E). Fibrillogenesis was blocked only in the transparent regions of the mask, while the opaque regions had protected FN from exposure, permitting subsequent cell-induced FN rearrangement. When using a photo mask featuring an optical density gradient, the degree of FN matrix remodeling could be gradually controlled (Supplementary Figure 8).

### Switching Off Cell-Mediated FN Fibrillogenesis During Live Cell Imaging

**FIGURE 3:**
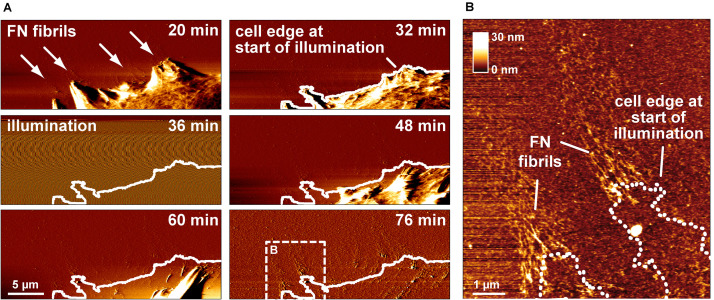
Switching off FN fibrillogenesis using visible light. **(A)** AFM deflection images extracted form an AFM timelapse series (Supplementary Movie 3). The AFM images display a retracting membrane region of a REF cells seeded onto unlabeled FN. Emerging FN nanofibrils become visible after 20 min. After 32 min the sample is transiently illuminated using a 20× lens and a standard fluorescence microscopy lamp without filter for 10 min. During illumination AFM imaging becomes unstable, but normalizes when the light source is turned off (48 min). A white line traces the membrane outline at the onset of illumination. **(B)** A higher resolution AFM height image of the dashed box in shown in panel **(A)** with a dotted line indicates the membrane edge at the beginning of illumination, demonstrating near instantaneous inhibition of fibrillogenesis when the light source was turned on.

The previous experiments demonstrated that cell-mediated FN fibrillogenesis never initiated if the FN layer had been pre-illuminated before cell seeding. We also explored whether already ongoing FN fibrillogenesis processes could be stopped by illumination. During continuing AFM scanning, the AFM imaging area was temporarily illuminated using a 20× lens and a standard fluorescence microscopy lamp for 15 min to test whether this would arrest active fibrillogenesis (Figure 3A). During the illumination phase, AFM imaging was transiently unstable, but normalized as soon as the light source was turned off. Tracing the cell outline at the onset of illumination showed that cell membrane retraction had continued unimpeded during and after illumination. In contrast, high-resolution AFM images showed that FN nanofibrils extended only up to the position of the cell edge at the beginning of illumination (Figure 3B), but not beyond, demonstrating near instantaneous inhibition of fibrillogenesis after the light source was turned on. Since cell membrane retraction continued during and after illumination, these experiments also verified that FN fibrillogenesis occurred simultaneously with membrane retraction, instead of pre-formed FN fibrils becoming uncovered during membrane retraction.

### Influence of Exposure Time and Illumination Wavelength

**FIGURE 4:**
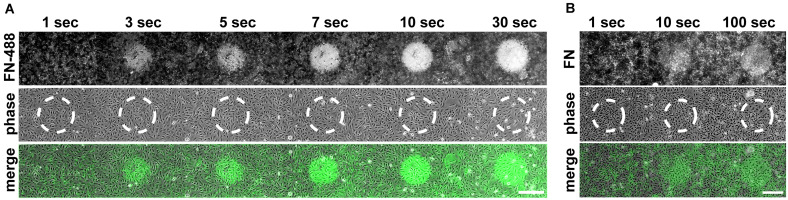
**(A)** Inhibition of FN fibrillogenesis as a function of exposure time. Circular areas (∼0.02 mm^2^ each) on the same FN-AF488 substrate were pre-exposed at a wavelength of 480 nm and a power density of ∼0.1 mW/mm^2^ for 1, 3, 5, 7, 10 or 30 s. MEFs were then cultured on the substrate in the dark for 16 h and subsequently fixed. FN-AF488 fluorescence images (upper row) show circular regions containing non-reorganized FN, which appear brighter in comparison to the darker areas containing reorganized fibrils. Phase contrast images of MEF cells (middle row) demonstrate homogeneous cell distributions over the whole sample, including the exposed areas (marked by dashed white circles). **(B)** Circular areas pre-exposed on unlabeled FN exposed for 1, 10 or 100 s. After MEF culturing (16 h) and fixation, FN was visualized by antibody staining (upper row). Again, bright circular regions demonstrate inhibition of fibrillogenesis in the pre-exposed areas. The lower contrast compared to the images in panel **(A)** is attributed to antibody staining of secreted cellular FN in addition to the surface-adsorbed plasma FN. Scale bars 50 μm.

Having established the inhibitory effect of visible light on cell-induced FN fibrillogenesis, we further investigated the dependence of this process on exposure time, illumination wavelength and a potential influence of fluorophore labeling. To study the inhibition of FN fibrillogenesis as a function of exposure time, circular areas (∼0.02 mm^2^) on FN-AF488 were pre-exposed at a power density of 0.1 mW/mm^2^ and a wavelength of 480 ± 20 nm for 1, 3, 5, 7, 10 or 30 s using a 63× objective. After culturing MEF cells on the pre-exposed substrates for 16 h and chemical fixation, overview fluorescence images were collected using a 20× objective (Figure 4A). First inhibitory effects, corresponding to circular areas of homogeneous, unmodified FN, became visible after as little as 3 s of pre-exposure, while full FN fibrillogenesis inhibition was achieved within ∼30 s of pre-exposure. The time-dependent increase in size and intensity of the unremodeled regions on the FN substrate indicated a dose-dependent irradiation effect. Corresponding phase contrast images (Figure 4A) again demonstrated a homogeneous cell distribution across the entire substrate independently of exposure area.

So far all light exposure experiments were performed using fluorescently labeled FN. Fluorophores are sites of increased energy adsorption, which can lead to local photo-damage in near-by proteins (DeRosa and Crutchley, 2002; Pattison et al., 2012). To test whether the labeling dye is responsible for the light-induced inhibition of FN fibrillogenesis, exposure-time dependent experiments were also performed using unlabeled FN. In this case the FN layer was visualized by immunofluorescence staining with a polyclonal anti-FN antibody after cell incubation (Figure 4B). The resulting fluorescence images again revealed circular patterns of unremodeled FN in pre-exposed areas, demonstrating that covalent fluorophore coupling is not required for light sensitivity of FN. Similar results were obtained when using alternative preparations of Human or Bovine plasma FN (Supplementary Figure 9). The contrast between exposed and unexposed regions on unlabeled FN was lower compared to experiments using labeled FN because the antibody staining also detected cellular FN secreted by the MEFs within the 16 h incubation time frame in addition to the substrate-adsorbed plasma FN. For unlabeled FN the inhibitory effect was not observed at exposure times <10 s, in contrast to a minimum exposure time of ∼3 s for labeled FN (Figure 4A). Thus, fluorophore labeling enhances the photo-sensitivity of FN molecules, but is not required for the photo-induced effect *per se*. Systematically varying the exposure wavelength between 400 and 640 nm demonstrated efficient inhibition of fibrillogenesis of unlabeled, AF488-, and AF568-labeled FN up to 560 nm, and partial or full loss of the inhibitory effect at longer wavelengths (Supplementary Figure 10).

### Testing Mechanical Properties of FN by AFM Scanning

**FIGURE 5:**
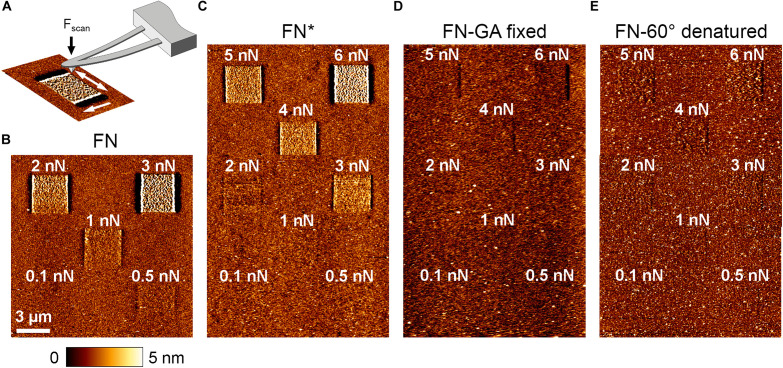
**(A)** Schematic depiction of FN rearrangement by AFM scanning. First, an overview scan (15 × 15 μm^2^) at minimal scan force (∼0.1 nN) verified homogeneous FN distribution. Sub-areas of 3 × 3 μm^2^ were then scanned on each sample with different scan forces ranging from 0.1 to 6 nN. Afterward an overview scan at minimal force (∼0.1 nN) was again performed to visualize potential FN rearrangement in the smaller scan areas. Scale bar 3 μm. **(B)** Untreated FN, **(C)** light-exposed, **(D)** glutaraldehyde-fixed, and **(E)** thermally denatured FN.

Cell-induced FN fibrillogenesis is a mechanical process during which individual FN molecules are extended and bundled into lager fibrillar structures as a result of intracellular forces transmitted by integrin receptors (Singh et al., 2010). The inability of cells to reorganize pre-exposed FN could therefore indicate light-induced alterations in mechanical properties of FN. We used AFM imaging at defined scanning forces to compare the mechanical properties of FN before and after light exposure in a cell-free system. Atomic force microscopy scanning is well-suited to investigate the mechanical stability of surface coatings. For instance, we previously studied the mechanical properties of different collagen matrices by scanning the substrate with increasing contact force (Friedrichs et al., 2007). In case of FN, elevated scan forces rearrange the FN layer into fibril-like structures, possibly involving partial extension/unfolding of FN molecules and fibrillar bundling (Figure 5A). The minimal scan force inducing structural rearrangement of FN consequently provides a measure of its mechanical stability. For scanning experiments, atomically flat mica substrates were coated with FN and scanned in PBS in contact mode. First, overview scans (15 × 15 μm^2^) using a minimal scan force of 0.1 nN verified homogeneous FN distribution. Afterward, five smaller (3 × 3 μm^2^) areas inside the overview region were scanned with increasing forces ranging from 0.1 to 3 nN. Finally, a second overview scan of the same region was performed, again using the minimal force of 0.1 nN to visualize potential FN rearrangement introduced during the preceding high force scans (Figure 5A). Unexposed FN showed fibrillar structures starting at a scan force of 0.5 nN (Figure 5B), while a scan force of 3 nN yielded maximal rearrangement. This scan force induced linear surface structures with a height profile (∼10 nm) similar to cell-induced FN nanofibrils (Gudzenko and Franz, 2015). In contrast, pre-exposed FN (FN^∗^, 9 mW/cm^2^, 10 min) showed clear rearrangement only at scan forces of 2 nN or higher, while full fibrillar induction and surface roughening required 6 nN (Figure 5C). These results demonstrated that exposed FN requires larger external forces for remodeling. Nevertheless, at sufficiently large scan forces (≥6 nN), FN rearrangement and surface roughening could be induced to the same extent as on non-exposed FN. In contrast, when artificial chemical crosslinks were introduced into non-exposed FN by glutaraldehyde fixation (Kiernan, 2000), no prominent fibrillar structures or surface roughening was induced even at the maximal scan force of 6 nN (Figure 5D) and FN fibrillogenesis was prevented completely. Because residual rearrangement of unfixed, exposed FN was still possible at elevated forces, exposure to light and chemical crosslinking apparently stiffen FN by different molecular mechanisms.

We also considered that exposure to highly focused, high-intensity light during fluorescence microscopy could lead to inactivation of FN due to thermal denaturation (Tanford, 1968). To test this possibility, we thermally denatured FN by incubation at 60°C for 30 min (Ingham et al., 1984) and afterward investigated the effect of force application on fibrillogenesis by AFM scanning. With increasing scan force, FN molecules were minimally shifted back and forth during scanning, leading a subtle structural changes (Figure 5E). However, regardless of scan force no fibrillar structures were induced, suggesting complete inability for fibrillogenesis of heat-denatured FN. Importantly, the complete lack of fibrillogenesis even at the maximal scan force of 6 nN was markedly different to exposed FN, which still displayed full remodeling capacity at high force. Thus, thermal denaturation causes complete inactivation of FN fibrillogenesis, while exposure to light only increases the required force threshold for fibrillogenesis. We therefore concluded that light-induced inhibition of fibrillogenesis is independent of thermal effects. In agreement, irradiation at longer wavelength (>600 nm) permitted cell-induced FN re-arrangement (Supplementary Figure 10), despite the higher sampler heating potential at this spectral range. Moreover, homogenous sample illumination on a cold light source (lightbox) inhibited FN fibrillogenesis efficiently, although this treatment induced no detectable change in temperature.

### Reducing the Impact of Light on the FN Fibrillogenesis by Removing Reactive Oxygen Species

**FIGURE 6:**
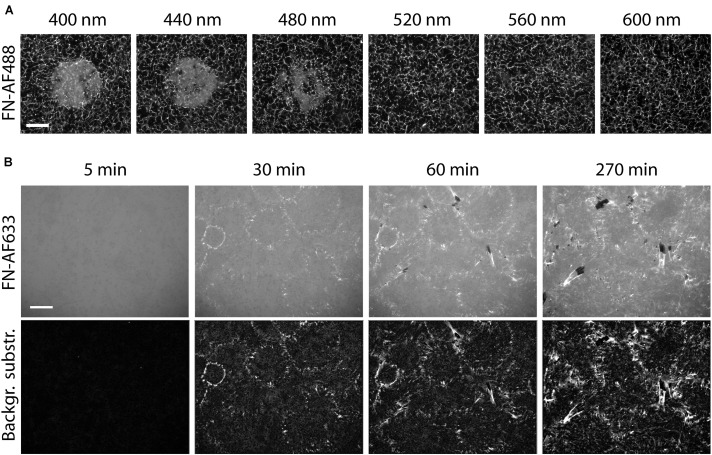
Reducing the photo damage to FN by removing ROS. **(A)** Circular areas on a FN-AF488 substrate in cell culture medium were pre-exposed at the indicated wavelengths ranging from 400 to 600 nm. The medium was supplemented with 0.5 U/ml oxyrase and 10 mM DL-lactate as a substrate. Scale bar 50 μm. **(B)** Reorganization of FN-AF633 by HFFs in DMEM medium containing 1% FCS, 1 mM MnCl_2_, 0.5 U/ml oxyrase and 10 mM DL-lactate. Fluorescence images of FN-AF633 (top row) taken after 5, 30, 60, and 270 min of incubation. Background fluorescence subtraction to increase image contrast and to improve fibril visibility (lower row). Scale bar 20 μm.

The previous experiments demonstrated that inhibition of FN fibrillogenesis by light depends on total light exposure dose (intensity, exposure time) and wavelength. Photo-dependent effects on protein structure and function are well-known and have been frequently linked to the light-induced production of reactive oxygen species (ROS). Exposure of aqueous solutions to high-energy light, such as the UVB (260-320 nm) or UVA (320-400nm) bands, causes production of ROS, including singlet oxygen (1O_2_), superoxide (O_2_^–^), its protonated form (hydroperoxyl radical; HOO^–^), hydrogen peroxide (H_2_O_2_) and the hydroxyl radical (HO^–^) (Burns et al., 2012). The creation rate of 1O_2_ is furthermore enhanced in presence of photosensitizers, i.e., molecules absorbing UV and visible light, such as organic dyes (DeRosa and Crutchley, 2002) or other chromophores (Pattison et al., 2012). Reactive oxygen species levels can be suppressed by adding biocatalytic oxygen-reducing agents, including enzymes such as oxyrase (Ho et al., 2003). To test whether ROS in the medium contribute to the light-dependent inhibition of fibrillogenesis, FN-AF488 was exposed in presence of 0.5 U/ml oxyrase in PBS at wavelengths ranging from 400 to 600 nm using a 63× objective. Afterward, PBS was replaced by DMEM medium and MEF cells were seeded on FN and incubated for 16 h. After cell fixation, overview images of exposed areas were taken with a 20× objective (Figure 6). The fluorescence images of FN-AF488 demonstrate inhibition of fibrillogenesis by exposure at wavelengths only up to 480 nm, in contrast to the corresponding experiments without oxyrase (Supplementary Figure 10B) which showed partial inhibition up to 640 nm. Moreover, at 480 nm the affected area was much smaller and had a smooth transition from the remodeled to unremodeled FN. Reduced inhibition of fibrillogenesis in presence of oxyrase therefore suggests that ROS play a main role in changing the properties of FN during illumination.

### Optimized Conditions for Visualizing FN Fibrillogenesis by Fluorescence Microscopy

Our results show that FN fibrillogenesis process cannot easily be observed by conventional fluorescence time-lapse imaging due to strong photo-induced inhibition effect. However, the presented results also provide strategies to minimize photo-damage of FN during time-lapse imaging. Since fibrillogenesis is strongly suppressed at wavelengths below 400 to 560 nm (Supplementary Figure 10), it would be beneficial to label FN with dyes that are excited at longer wavelengths. For instance, Alexa Fluor 633 is excited at a wavelength of 632 nm, potentially minimizing the negative effect of the excitation with shorter wavelength light. Furthermore, removing ROS from the medium by adding oxyrase substantially reduces the impact of light on FN (Figure 6A). To further minimize photo damage, images should be collected at low frequency (one image every 30 min). Furthermore, integrin affinity to FN can be enhanced and FN fibrillogenesis by adding 1 mM Mn_2__+_ to the imaging medium (Edwards et al., 1988; Mould et al., 1995). Using a combination of these strategies, FN fibrillogenesis induced by HFF cells could be observed by fluorescence time-lapse microscopy for the first time for a total duration of 270 min, albeit with comparatively low time resolution (Figure 6B and Supplementary Movie 4). Small fibrils are distributed over the entire imaged region as early as 30 min after cell seeding. At this time, the mean fibril length is 0.6 ± 0.2 μm and the mean velocity of fibril formation is 37 ± 9 nm/min. This value is lower than the velocity obtained by AFM time-lapse imaging (157 ± 107 nm/min; Gudzenko and Franz, 2015). A reason might be the different temporal and spatial resolutions between the fluorescence and AFM time-lapse. Atomic force microscopy images were taken at a much higher rate (every 3 min) and with nm resolution, so that the fibril dynamics can be analyzed more precisely. Furthermore, according to the fluorescence images taken 60 and 90 min after cell seeding, the fibril growth rate decreases to 13 ± 5 nm/min at later time points. Starting after 60 min, and becoming more clearly after 270 min, cells start to remove FN fibrils from the surface, leaving dark, FN-free areas behind.

### Using Light-Dependent Stiffening of FN to Investigate Mechano-Sensitive Aspects of Focal Adhesion Function

**FIGURE 7:**
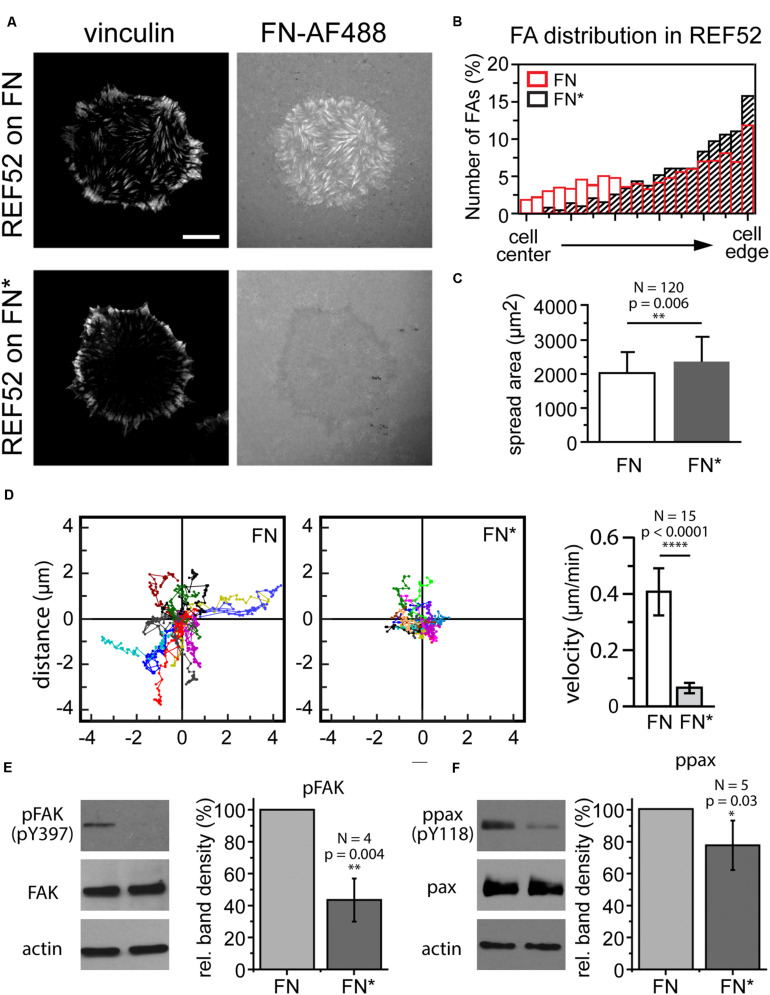
**(A)** Focal adhesion distribution in REF cells incubated on unexposed (FN) or exposed FN (FN^∗^) for 4 h. Focal adhesions visualized by vinculin staining. Bar 10 μm. **(B)** Comparison of focal adhesion localization between cell edge and center. **(C)** Cell spreading area (mean ± SD) on unexposed and exposed FN. Statistics: unpaired *t*-test, *N* = 120, *p*-value 0.006. **(D)** Cell scatter plots and cell migration velocity (mean ± SD) of REF cells on unexposed and exposed FN. Statistics: unpaired *t*-test, *N* = 15, *p*-value, *p* < 0.0001). Western blot analysis of panels **(E)** FAK and **(F)** paxillin phosphorylation after incubation on unexposed and exposed FN. Actin bands are loading controls. For each experiment the intensities of the phosphorylated bands were normalized to the respective total FAK or paxillin protein levels, and averaged relative FAK or paxillin phosphorylation levels plotted as mean ± SD. Statistics pFAK: one-way *t*-test, *N* = 4, *p*-value = 0.004, ppax: one-way *t*-test, *N* = 5, *p*-value = 0.03.

The photo mask patterning experiments (Figure 3) demonstrated that visible light can be used as to selectively adjust the mechanical properties of a FN layer and to locally control cell-induced fibrillogenesis. Finally, we investigated whether light-dependent adjustment of FN mechanics could in turn be used to delineate mechanosensitive signaling pathways and potential changes in cell behavior dependent on functional FN fibrillogenesis. First, we assessed how externally blocking FN fibrillogenesis affected cell morphology, focal adhesion site arrangement and cell migration. Rat embryonic fibroblasts cells seeded on unexposed FN for 1 h displayed large focal adhesions at the cell periphery and a more uniform distribution of smaller focal adhesions across the entire basal cell side (Figure 7A). Cell-induced fibrillar FN patterns largely overlapped with focal adhesion arrangement, demonstrating active FN fibrillogenesis at focal adhesions throughout the basal cell side. In contrast, focal adhesions formed predominantly at the cell periphery (Figures 7A,B) when FN fibrillogenesis was blocked by pre-illumination (Figures 7A,B). These results are consistent with previous findings that focal adhesions on FN first form at the cell periphery and then translocate centripetally while pulling the underlying FN layer into fibrils as a result. Cells on exposed FN displayed slightly enhanced mean spreading areas (Figure 7C) but drastically reduced migration speeds (Figure 7D), suggesting that functional fibrillogenesis is required for normal cell movement and focal adhesion rearrangement, but not cell spreading. Finally, we investigated cellular signaling transduction pathways possibly affected by blocked FN fibrillogenesis. Focal adhesion kinase (FAK) is a non-receptor protein tyrosine kinase co-localizing with integrins in focal adhesions and a key member of the integrin-dependent signaling pathways (Zachary and Rozengurt, 1992). Upon integrin engagement at the cell surface FAK becomes activated and phosphorylated on multiple sites, including the Y397 autophosphorylation site, which then serves as a binding site for a number of signaling partners such as c-Src and PI3-kinase (Cobb et al., 1994; Sabe et al., 1994; Guinebault et al., 1995; Schlaepfer and Hunter, 1997). Paxillin is a multifunctional focal adhesion adapter protein which also becomes tyrosine phosphorylated upon integrin-dependent cell adhesion to extracellular matrix proteins (Burridge et al., 1992). In particular, phosphorylation on tyrosine 118 has been linked to the regulation of focal adhesion turnover and cell migration (Iwasaki et al., 2002; Petit and Thiery, 2000) and is negatively regulated by mechanical force (Zaidel-Bar et al., 2007). Western blot analysis showed that inhibiting FN fibrillogenesis reduces FAK (Y397) and paxillin (Y118) phosphorylation levels by 43 ± 14% and 77 ± 15%, respectively (Figures 7E,F). These experiments suggest that light-induced stiffness modulation of FN layers could become a useful future tool to delineate mechanosensitive signaling pathways underlying integrin-mediated FN remodeling.

## Discussion

### FN Requires Special Fluorescence Microscopy Imaging Conditions Due to Its Photo Sensitivity

In this study we describe an unexpected inhibitory effect of visible light on the cell-induced fibrillogenesis of human plasma FN. Even comparatively mild illumination conditions commonly used in life-cell TIRF or epi-fluorescence microscopy (milliseconds exposure times, laser powers in the low milliwatt range) completely prevented fibrillar aggregation within the focal area, while fibrillogenesis outside the focal area was unaffected. The photo sensitivity of FN may have important implications for many cell biological and biofunctionalization experiments, since surface coating with plasma FN is widely used for enhancing cell attachment and for studying integrin-dependent cell spreading and migration behavior. While both non-exposed and pre-exposed FN supported cell spreading, we observed large changes in cell migration speed, focal adhesion organization and mechano-sensitive signaling pathways involving FAK and paxillin phosphorylation on pre-exposed FN. Thus, cell migration and signaling experiments can yield different results depending on whether they are performed on non-exposed FN, for instance inside dark cell culture incubators, or during live-cell fluorescence imaging.

The strong light-sensitivity of FN initially prevented us from observing early steps of FN fibrillogenesis by fluorescence microscopy, even when minimizing exposure intensity and time. However, the severity of the inhibitory effect could be reduced by illumination with longer wavelengths and by removing ROS from the imaging solution. Besides minimizing exposure time and intensity, strategies for fluorescence imaging of plasma FN should therefore include choosing fluorescent dyes with long excitation wavelength. Using Alexa Fluor 633 dye (excitation wavelength 632 nm) for FN labeling and adding enzymes such as oxyrase to remove ROS from the medium allowed us to observe fibrillogenesis for up to several hours, albeit at low frame rates to reduce the total exposure dose. Using fluorophores at even longer excitation wavelength may further aid FN imaging. On the other hand, AFM timelapse imaging avoids light-induced effects altogether and high-speed AFM scanning (Yamamoto et al., 2010) may be a more suitable method to study early FN fibrillogenesis at greatly enhanced temporal resolution.

### AFM Imaging at Elevated Scan Forces as a Useful Tool to Assess FN Matrix Stiffness

AFM scanning at increasing scan forces proved to be a useful tool to assess the mechanical properties of FN. Although high tip forces induced similar surface roughening of the FN layer as cell-induced FN fibrillogenesis (height change ∼10 nm), it is no clear if similar molecular changes underlie both processes. Importantly, at sufficiently high scan forces, pre-exposed FN could be remodeled to the same extent as non-exposed. Illumination therefore does not abolish the capability of FN for fibrillar remodeling altogether, but apparently raised the force threshold for this process, equivalent to a mechanical stiffening of the FN layer. This force threshold was above the traction forces different fibroblast cell lines can exert on the FN layer, as remodeling of pre-exposed FN was never observed at the basal cell side. Exposure to light could, for instance, enhance the mechanical stability of the FN layer through the introduction of additional intra- or inter-molecular covalent cross-links, stabilizing FN in its globular, folded conformation and preventing unfolding and subsequent fibril formation.

We initially considered thermal effects induced by illumination as a possible reason underlying the loss of the ability for fibrillogenesis. Irradiation with light at the red edge of the visible spectrum (near-infrared) and infrared light (>700 nm) may induce photo-thermal damage and heat denaturation of proteins, including FN. Studies on FN fragments (Vuento et al., 1980; Odermatt et al., 1982; Litvinovich et al., 1998) as well as whole FN molecules (Ingham et al., 1984; Pauthe et al., 2002) demonstrated that FN is thermally stable between 4 and 60°C, fully recovering its specific conformational state after re-equilibration at 20°C (Nelea et al., 2008). Above 60°C, thermal denaturation of FN begins and is typically completed within 30 min or less (Ingham et al., 1984). In agreement, FN heat-denatured at 60°C never showed fibrillar remodeling even at the highest scan forces. However, the AFM scan force experiments revealed different mechanical properties of thermally denatured FN compared to exposed FN, which retained the capacity for mechanically induced re-organization, albeit only at higher applied forces compared to non-exposed FN. Moreover, inhibition of fibrillogenesis was more effective at shorter wavelength, and absent at near-infrared wavelengths which potentially induce stronger sample heating, suggesting heat-independent mechanisms for the observed FN stiffening.

### Possible Photo Damage Targets in FN

While the precise light-triggered molecular changes responsible for the inhibition of FN fibrillogenesis established by our study remain unknown, the crucial role of ROS in this process points to photo-oxidation mechanisms (Murphy and Davidson, 2012). These could either directly target FN molecules, or acting via surrounding water or other molecules in the cell culture medium. Two general major pathways leading to photo-induced damage of proteins have been described (Bensasson, 1983; Pattison et al., 2012). In the first pathway mediated by UVB irradiation (280–315 nm), irradiation energy is directly absorbed by different amino acid residues (e.g., tryptophan, tyrosine, phenylalanine, histidine, methionine, cysteine, and cysteine disulfide bonds), resulting in the formation of electronically excited states and photo-ionization reactions. However, this pathway appears irrelevant to our FN experiments because we observed inhibition at visible wavelengths (400–640 nm). A second pathway involves the absorption of energetically lower light in the UVA (315–390 nm) and visible (390–700 nm) ranges by different photosensitizing components (Pattison et al., 2012), such as porphyrins (Afonso et al., 1999) and polyaromatic compounds (Phillips, 2010). Proteins can be cleaved by photo-inducible fragmentation in the presence of such sensitizers (Michaeli and Feitelson, 1994; Davies, 2003; Pattison et al., 2012). For instance, it was shown that lysozymes undergo photolysis in presence of O_2_ by tryptophan oxidation and radical formation (Hawkins and Davies, 2001). However, SDS-Page analysis indicated no FN fragmentation after irradiation (Supplementary Figure 11).

Dye molecules used for protein labeling form a further important group of potential sensitizers. In general, sensitizers are excited into a short singlet state and then either decay to the ground state while emitting light (fluorescence) or to the more stable triplet state, allowing reactions with surrounding molecules (Phillips, 2010). Energy transfer from sensitizers to surrounding proteins or water molecules can then result in the formation of ROO, RO, O_2_^–^ or OH radicals (Balasubramanian et al., 1990) or in the creation of singlet oxygen (^1^O_2_) from molecular oxygen (Pattison et al., 2012). These highly reactive molecules may oxidize other molecules in the immediate vicinity of the initially excited sensitizer. However, while labeling with Alexa Fluor dyes increased the sensitivity of FN to photo-inhibition of fibrillogenesis, it was not the dominant mechanism, as unlabeled FN was also affected.

Furthermore, photo-oxidation of proteins can result in irreversible cross-linking by the formation of intermolecular covalent bonds (Dean et al., 1984; Wolff and Dean, 1986; Bedwell et al., 1989), disulfide bond linkages through free thiol groups or via non-disulfide cross-linking pathways such as dityrosines (Mahler et al., 2009). In turn, existing di-sulfide bonds may also be targets of photo damage. Fibronectin contains a large number of intra-chain disulfide bonds, two inter-chain disulfide bonds (Petersen et al., 1983) and two sulfhydryl (SH) groups per monomer (Smith et al., 1982), and the disulfide bridges in FN are necessary for its biological activity (Ali and Hynes, 1978). Cysteine photo-oxidation via ^1^O_2_ results in the formation of cysteic acids (RSO_3_H) (Pattison et al., 2012). Photo-oxidation of disulfide bonds via electron transfer from sensitizers yields disulfide radical anions (RSSR^–^), which can rapidly dissociate into thiyl anion (RS^–^) and thiyl radical (RS) or transfer electrons to O_2_, creating O_2_^–^ (Creed, 1984). However, due to the size and complexity of the molecule, the irradiation influence of FN is still only incompletely understood and requires further studies.

## Conclusion

Time-lapse fluorescence microscopy imaging frequently provides important insights into dynamic biological processes. In the case of FN fibrillogenesis, however, a standard imaging approach cannot be applied due to severe photo-sensitivity of the FN molecule. Instead, successfully observing of fibrillogenesis by fluorescence microscopy requires a modified approach consisting of (1) minimizing exposure time and intensity, (2) using labeling dye with long excitation wavelengths (>600 nm), and (3) minimizing the ROS concentration in the imaging medium. On the upside, selective illumination of FN presents itself as a useful experimental tool to specifically turn off one of its hallmark functions – the ability to be remodeled into elastic fibrillar networks – while retaining other functions, such as promoting cell spreading and proliferation. Such an approach may be useful, for instance, for delineating mechanosensitive signaling pathways activated during integrin-mediated FN remodeling at focal adhesions. Since pre-exposed FN layers remained resistant to cellular remodeling for at least up to 3 days, pre-exposure by visible light may also be useful for making FN-biofunctionalized surfaces more durable. Lastly, given the wide-spread use of purified plasma FN for surface functionalization in cell biology experiments and biotechnology applications, light-dependent effects on FN function and, as a results on cell behavior, must be taken into consideration when comparing results from live-cell time-lapse experiments and experiments performed in dark tissue culture incubators.

## Data Availability Statement

All datasets generated for this study are included in the article/Supplementary Material.

## Author Contributions

CF conceived the study, performed live-cell AFM experiments, prepared figures, and drafted the manuscript. TG performed AFM and light microscopy experiments, carried out image and data analysis, prepared figures, and helped drafting the manuscript. All authors contributed to the article and approved the submitted version.

## Conflict of Interest

The authors declare that the research was conducted in the absence of any commercial or financial relationships that could be construed as a potential conflict of interest.

## Footnotes

1 www.lifetechnologies.com

2 www.wpiinc.com

3 www.agilent.com

4 www.zeiss.de

5 www.excelitas.com

6 https://www.bdbiosciences.com/en-eu

7 www.sigmaaldrich.com

8 www.olympus.com
