# Supplementary material for: Controlling Fibronectin Fibrillogenesis Using Visible Light
Source: Front Mol Biosci. 2020 Jul 8;7:149. doi: 10.3389/fmolb.2020.00149 (PMC7360794; doi:10.3389/fmolb.2020.00149)
Supplement: Supplementary file 1 [file Table_1.DOCX]

Supplementary Material

# Supplementary Figures

**
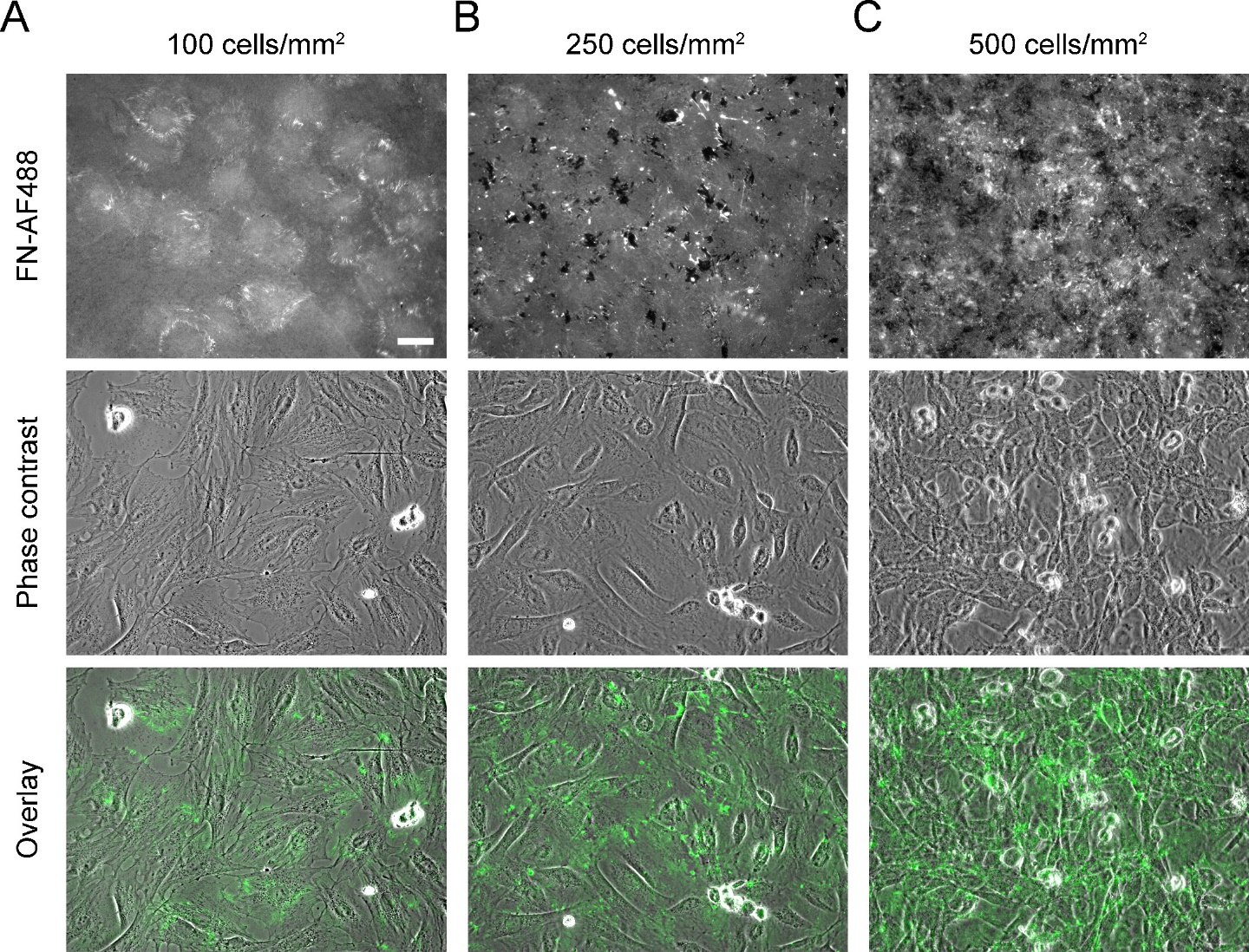
**

**Supplementary Figure 1.** MEFs incubated on Alexa Fluor 488-labeled fibronectin (FN-AF488) for 4 h and fixed for fluorescence microscopy (top panels) and phase contrast microscopy (middle panels), and merged images (bottom row). Scale bar 20 µm.

**
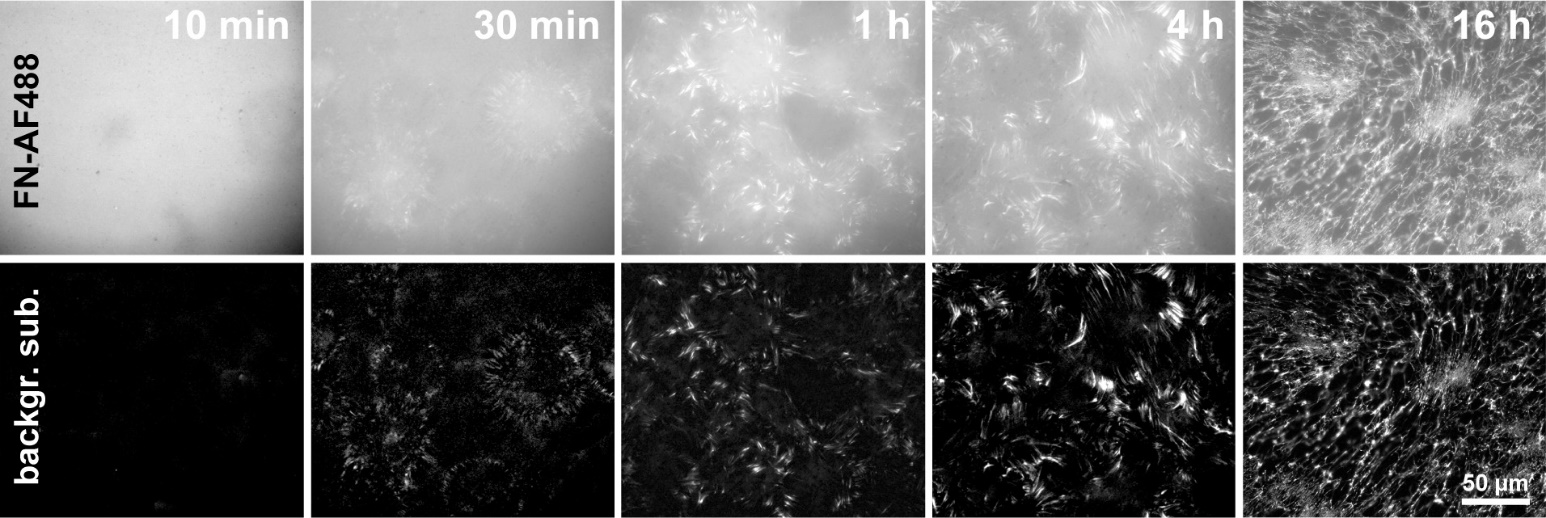
**

**Supplementary Figure 2.** MEFs incubated on FN-AF488 for 10, 30, 60 min or 16 h and fixed to visualize different stages of FN fibrils creation (upper row). Fluorescence image background subtraction for enhanced fibril visualization at different stages of fibrillogenesis (lower row).


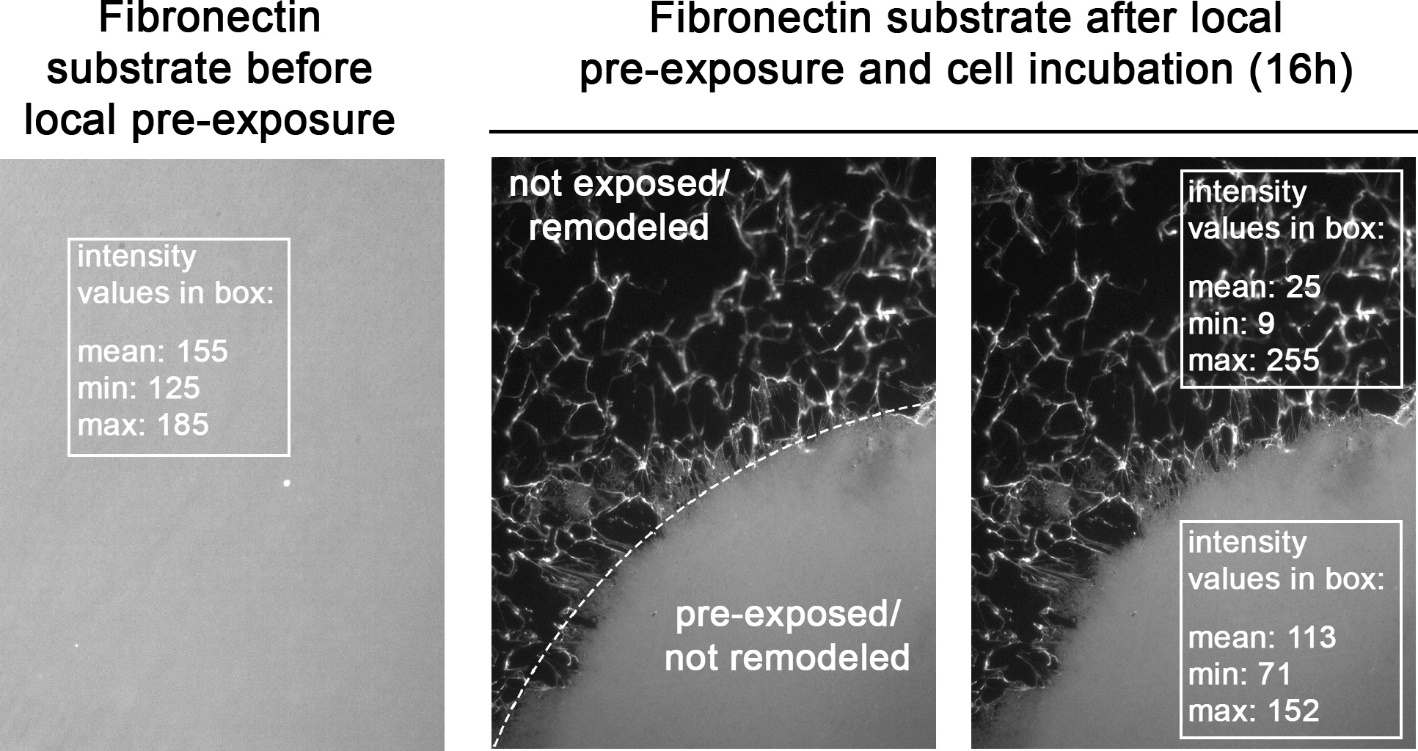


**Supplementary Figure 3.** Analysis fluorescence intensities of a homogeneous FN-AF488 substrate before illumination (left panel) and after pre-exposure of a circular area for 10 min and MEF cell incubation for 16h. (middle and right panel). The dashed line in the middle panel indicates the border of the pre-illuminated region. Boxes denote regions in which mean, maximum and minimum fluorescence intensity values were determined. Pre-exposed, unremodeled regions largely retain their mean fluorescence intensity values (except for a slight decrease due to photobleaching of the FN-AF488 dye during pre-exposure. Pre-exposed areas feature no fibrillar structures. In contrast, non-exposed, remodeled areas feature a wide distribution of intensity vales, ranging from near black values in areas of FN removal from the substrate, to fully saturated values in areas of tight fibrillar bundling. However, on average remodeled areas appear darker compared to unremodeled areas.


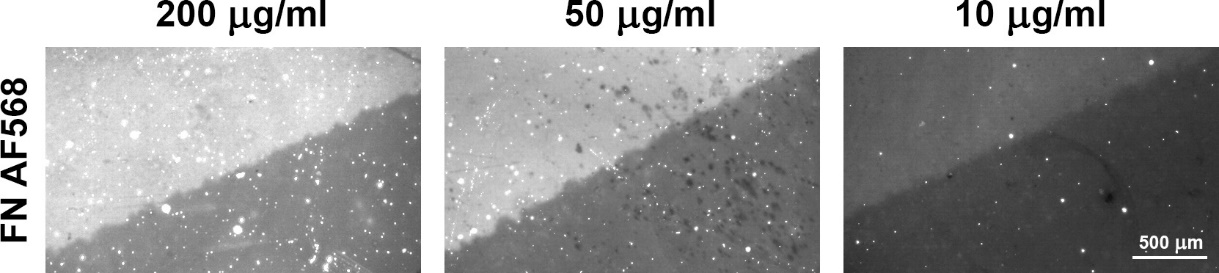


**Supplementary Figure 4.** Light-dependent inhibition of FN fibrillogenesis using different surface coating concentrations. After coating for 1h at the indicated concentrations, FN-AF568 substrates were pre-illuminated for 10 min using a stripe photo mask. Afterwards a confluent layer of MEF cells was seeded and incubated for 16 h. Images were collected using a 5x lens. Bright areas correspond to pre-exposed regions.


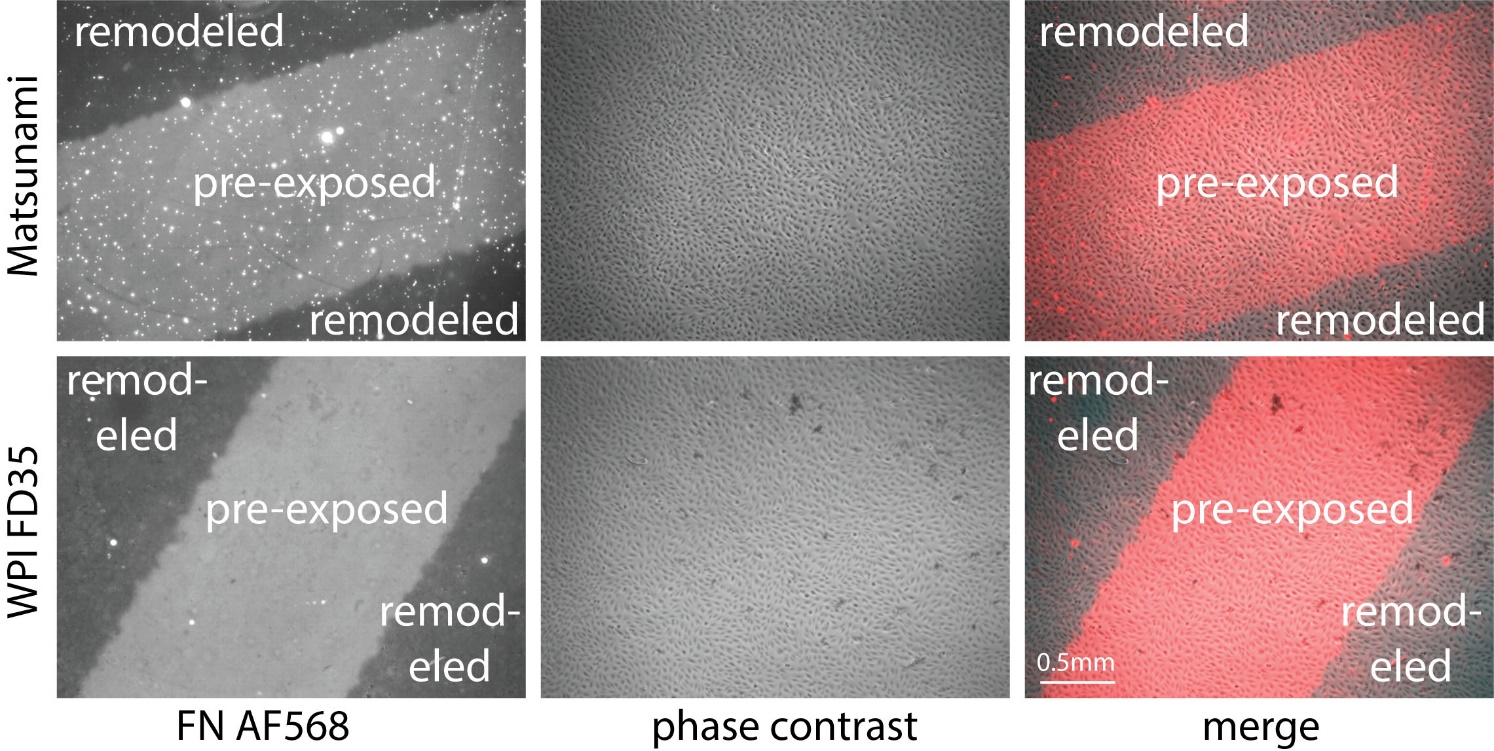


**Supplementary Figure 5.** Light-dependent inhibition of FN fibrillogenesis on different glass types. After coating for 1h at a concentration of 50μg/ml, FN-AF568 substrates (Matsunami, top row, and WPI FD35, bottom row) were pre-illuminated for 10 min using a stripe photo mask. Afterwards a confluent layer of MEF cells was seeded and incubated for 16 h. Fluorescence images were collected using a 5x lens. Bright areas correspond to pre-exposed areas featuring unremodeled FN. Bright spots correspond to AF568 aggregates.


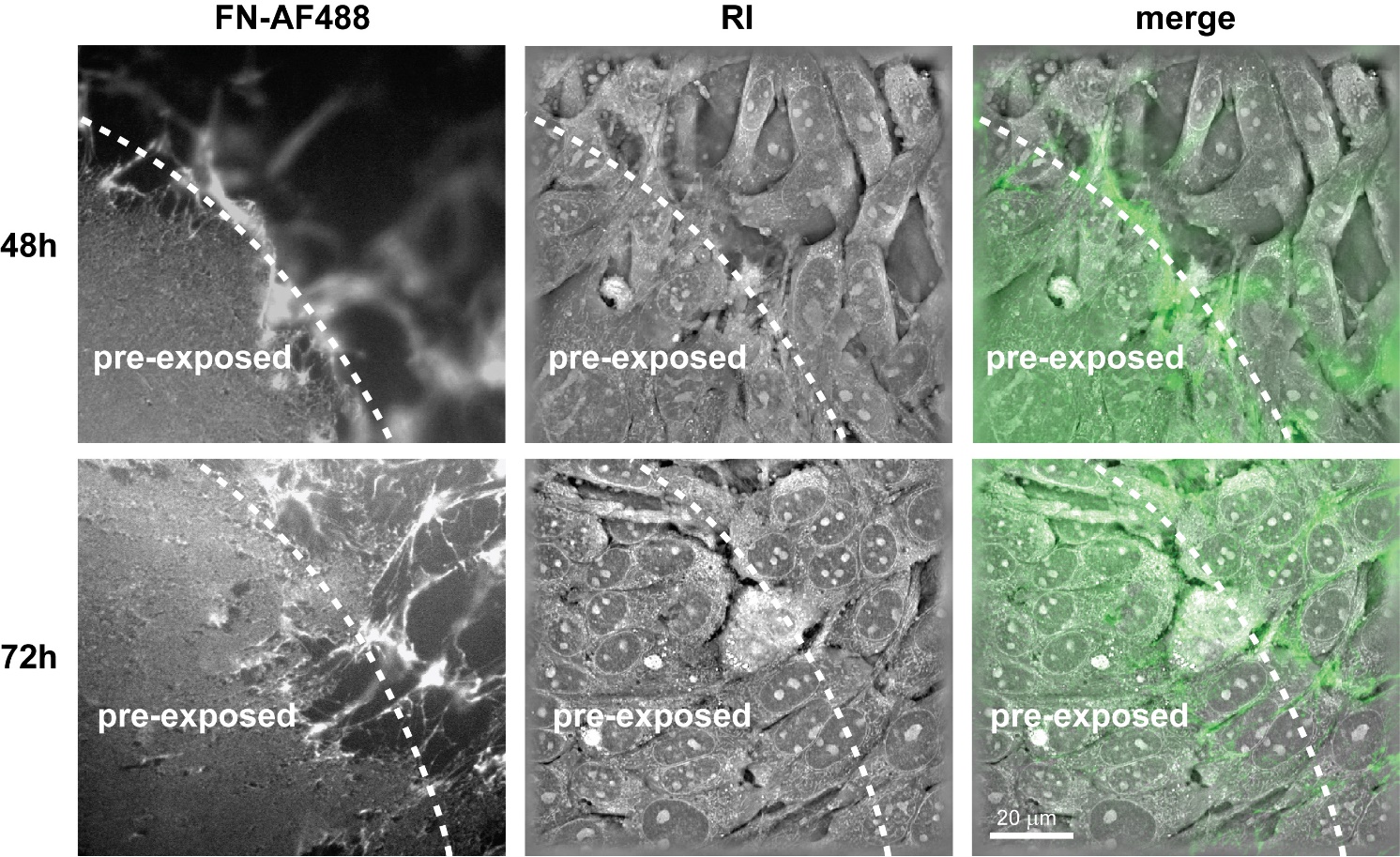


**Supplementary Figure 6.** Incubating MEFs for 48 or 72h on FN-AF488 locally pre-exposed through an Olympus 63x oil immersion lens for 10 min at full power of an X-Cite 120 illumination system. Fluorescence (middle panels) and holo-tomographic (right panels) images displaying differences in refractive index (RI) of living MEFs in DMEM were collected using a Nanolive 3D Explorer equipped with a heated sample chamber (Okolab) at 37ºC and 5% CO_2_. The dashed line indicates the edge of the pre-exposed area on the fibronectin substrate.

**
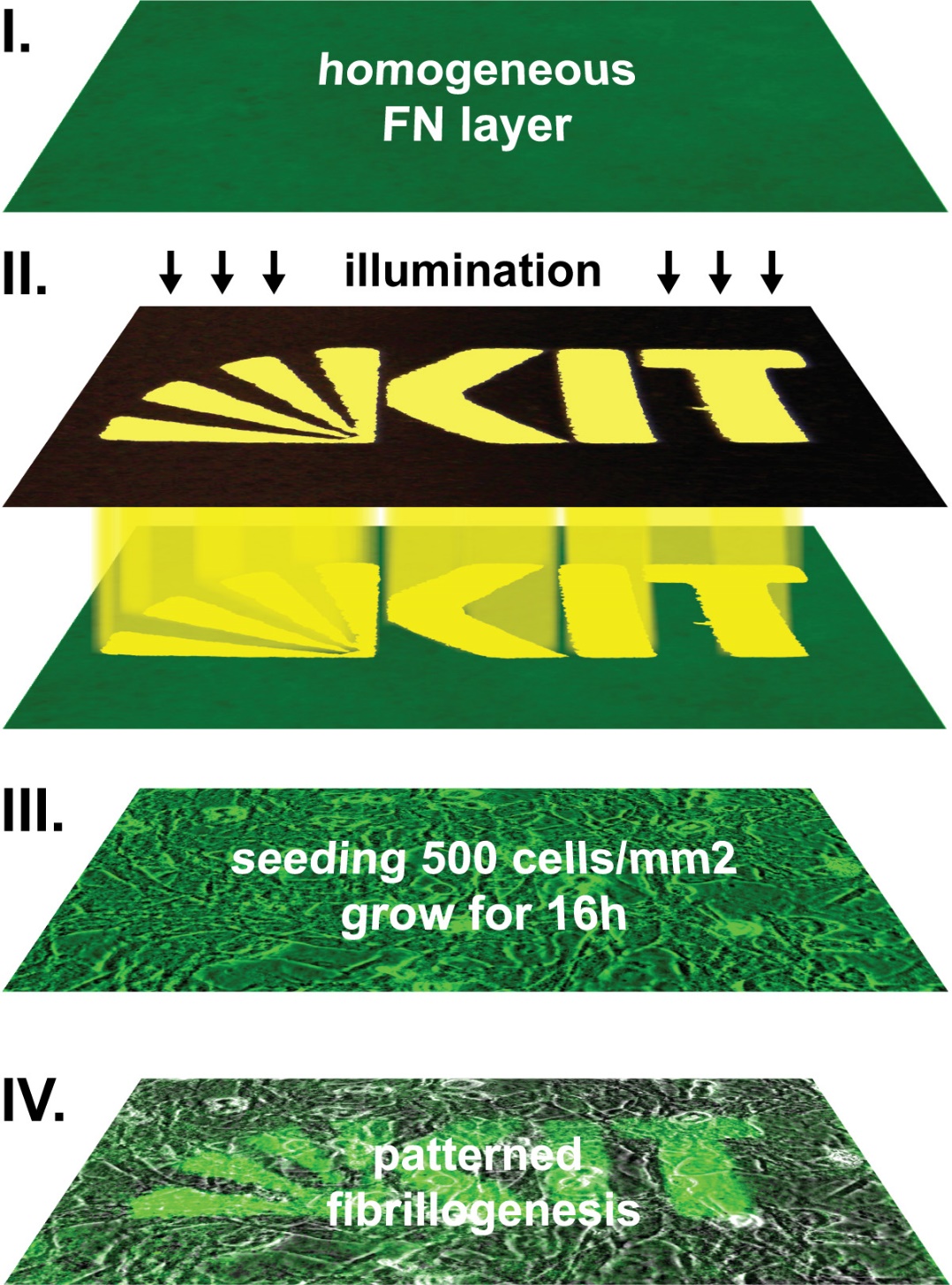
**

**Supplementary Figure 7.** Schematic depiction of the FN photo patterning process. **(I.)** A homogenous FN layer (either unlabeled or fluorescent-labeled) is adsorbed onto a glass substrate. **(II.)** The FN layer is exposed to visible light through a photo mask. **(III.)** Afterwards cells are seeded at high density (500 cells/mm^2^) and incubated on the pre-exposed FN layer in the dark for 16 h. **(III.)** Areas corresponding to the pre-exposed regions contain homogeneous, non-remodeled FN due to inhibited fibrillogenesis, while the remaining FN areas have been cooperatively remodeled into a 2-dimensional fibrillar FN network by cells.


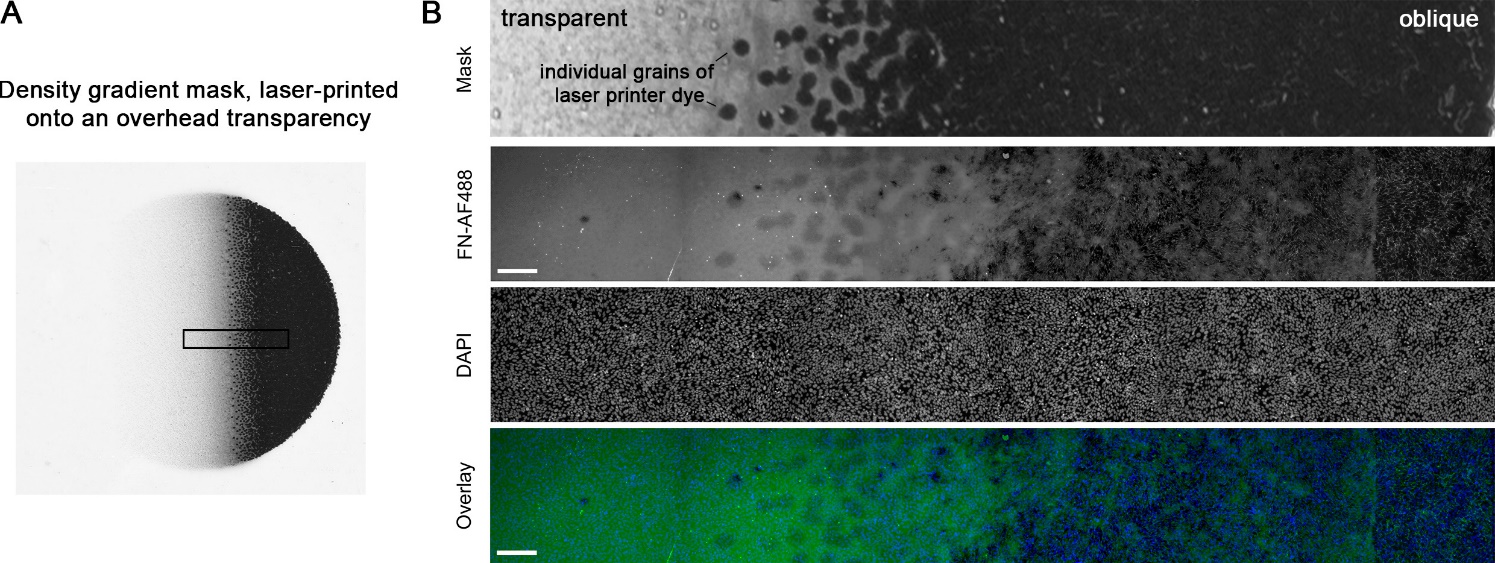


**Supplementary Figure 8.** Guiding FN fibrillogenesis using a graded photomask. **(A)** FN-AF488 adsorbed to FD35 glass bottom dishes at 50μg/ml for 1 h was exposed for 10 min at full power using an X-Cite 120 illumination system through a gradient mask (upper image) printed on transparent film. **(B)** An area of the FN substrate corresponding to the box in **(A)** was imaged after cell culturing, fixation and nuclei staining with DAPI. The fluorescence image of FN-AF488 (second row from top) demonstrates that the fibrillogenesis process was blocked in a dose-dependent manner below the transparent or semi-transparent regions. DAPI staining (second row from bottom) verifies a homogeneous cell distribution throughout the imaged region. The overlay image depicts the DAPI (blue) and the FN (green) channel. Scale bar 200 μm.


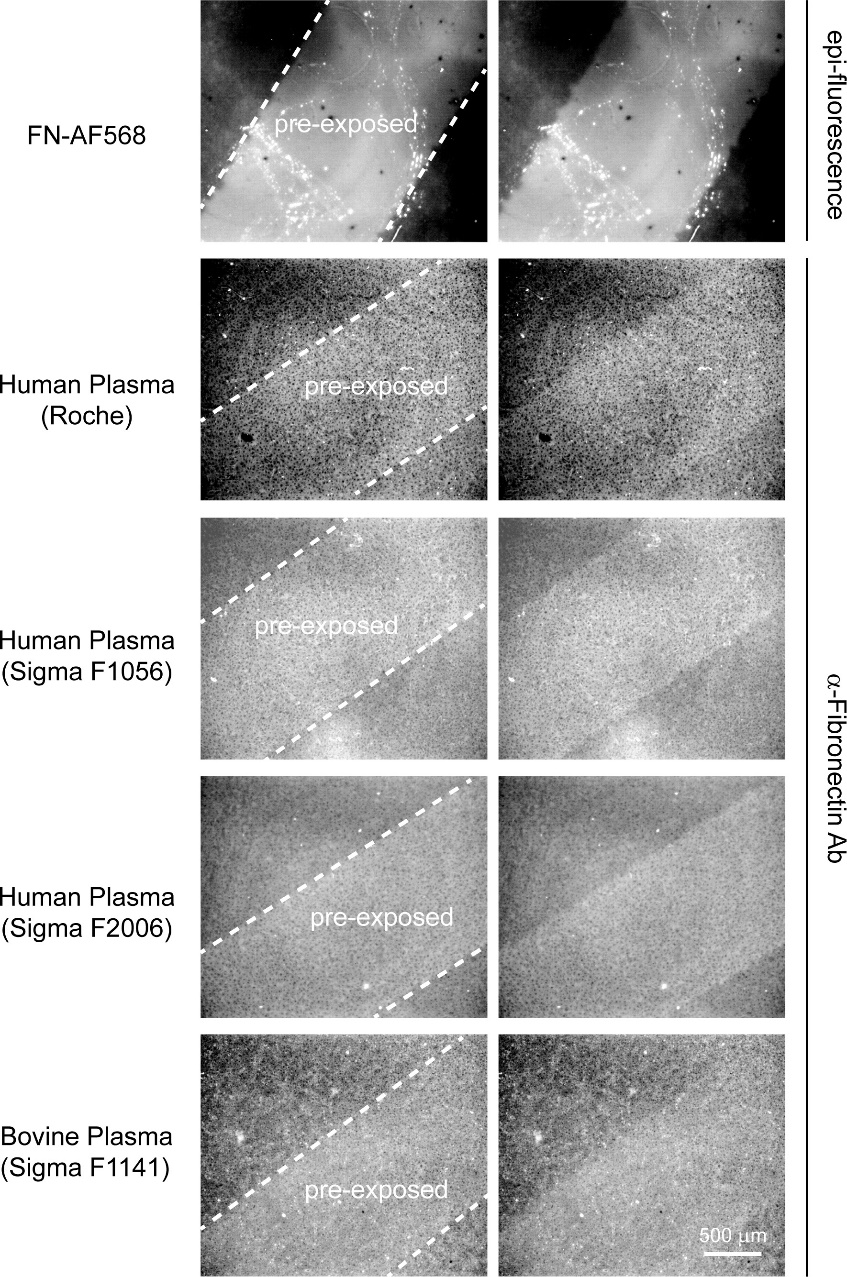


**Supplementary Figure 9.** Light-dependent fibrillogenesis inhibition of labeled and unlabeled fibronectin. Different human (Roche, Sigma F1056, F2006) and bovine plasma fibronectin (F1141) samples, as well as Alexa Fluor 568-labeled fibronectin (Roche) were used to coat glass bottom dishes (WPI FD35) at a concentration of 50 μg/ml for 1h in the dark. Afterwards, substrates were illuminated through a stripe mask for 10 min using a X-Cite illumination system. After seeding and growing a confluent monolayer of MEF cells for 16h and fixation, samples were imaged by epifluorescence (FN-AF568) or after staining for fibronectin using a rabbit polyclonal anti-fibronectin antibody (Sigma F3646) at 1:500, and a secondary anti-rabbit IgG antibody coupled to Alexa Fluor 568 (Life Technologies) at 1:100 using a 5x lens. The dashed lines in the left panel indicate the location of the mask. Regions that were protected from light by the mask show up as bright stripes due to partial or full suppression of fibrillogenesis. Contrast in the unlabeled samples between exposed and non-exposed region is lower compared to the FN-AF568 sample, since the polyclonal antibody recognizes both the adsorbed plasma and cellular fibronectin produced by the MEF cells.


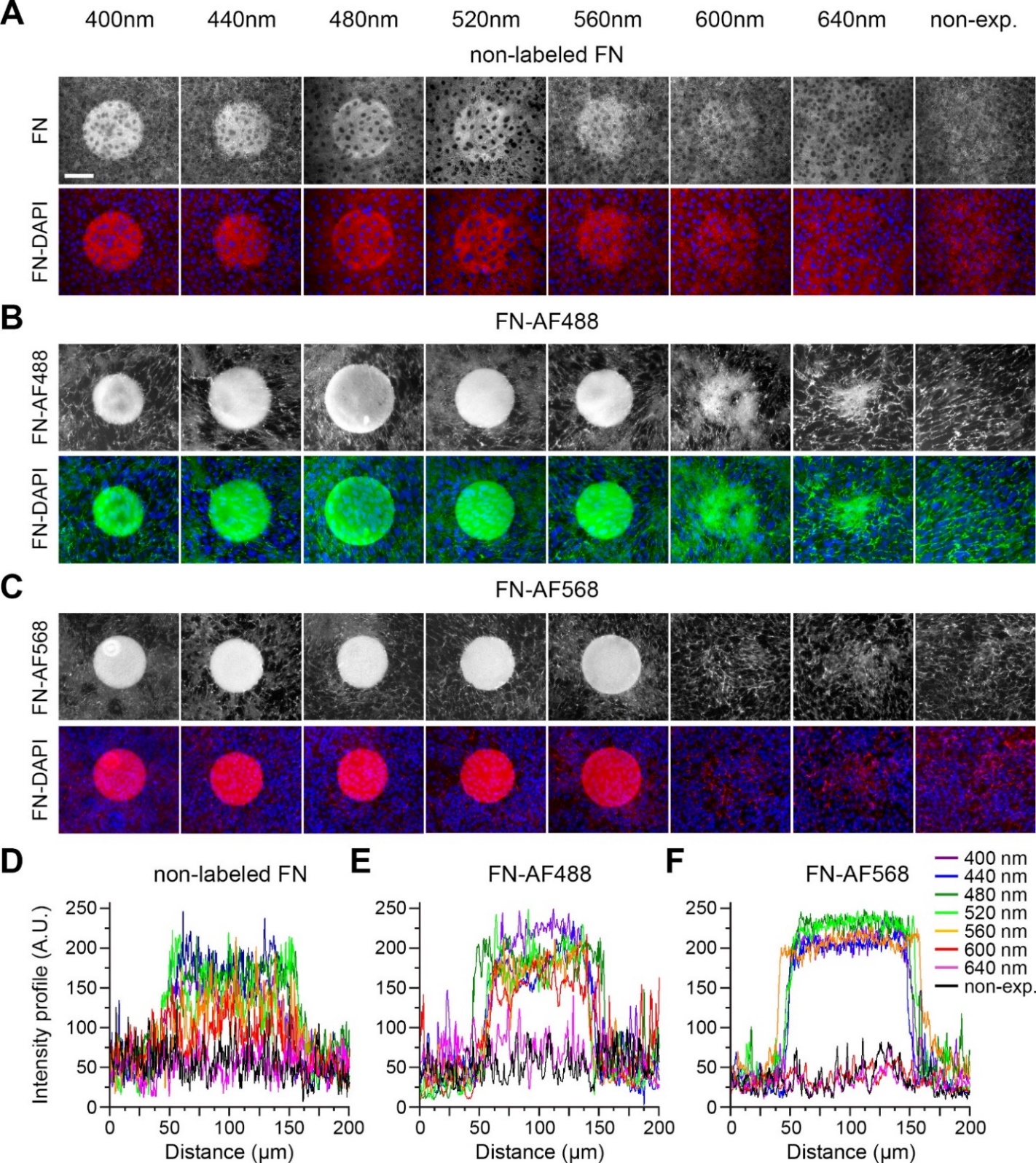


**Supplementary Figure 10.** Inhibition of fibrillogenesis as a function of wavelength. Equal circular areas (0.02 mm^2^) on unlabeled, FN-AF488- an FN-AF568 surfaces were illuminated through a LD Plan-Neofluar 63x lens (numerical aperture 0.75) with different wavelengths ranging from 400 to 640 nm in 40 nm steps using a tunable monochromator light source (Polychrome 5000 with a 150 W Xenon lamp). After culturing MEFs for 16 h in the dark, samples were fixed and DAPI stained. Unlabeled FN was also stained with a polyclonal anti-FN antibody (Sigma F3648). Fluorescence images were collected using a 20x lens. (A) Unlabeled FN, (B) FN-AF488, and (C) FN-AF568. Scale bar 50 µm. (D, E, F) Intensity profiles extracted from the corresponding fluorescence images (mean values from three independent experiments for each condition).


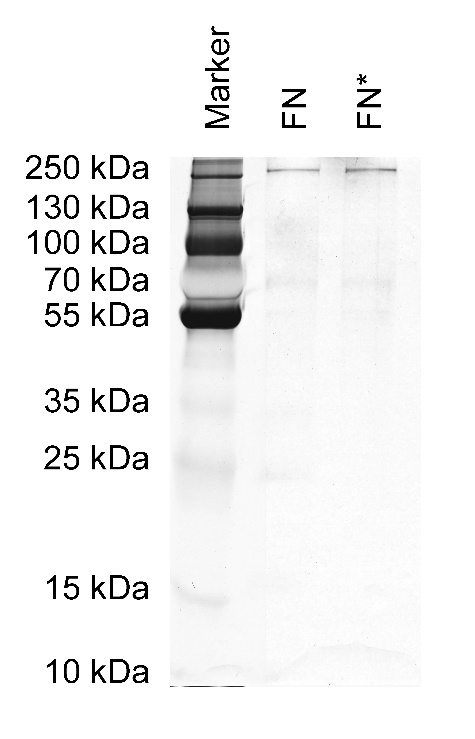


**Supplementary Figure 11.** Analysis of FN by SDS-PAGE. FD-35 dishes were covered with 50µl unlabeled human plasma fibronectin (Roche, 1 mg/ml) and incubated for 1h at room temperature. Samples were then washed two time in PBS and overlaid with 2ml PBS each. One sample (denoted by FN*) was exposed to light using an X-Cite 120 illumination system at full power for 1h, while the control sample (FN) was kept in the dark. After removal of PBS, samples were solubilized in 100 µl Tx-100 lysis buffer (1% Triton X-100, 50 mM Tris-HCl, 150 mM NaCl, 5 mM EDTA), scratched from the glass surface and centrifuged for 5 min at 13000 rpm. 5x loading buffer (5% SDS, 250 mM Tris-HCl (pH 6.8), 50% glycerol, 0.2% bromophenol blue, 500 mM DTT) was added (2.5 µl per 10 µl of sample), and samples were heated to 95°C for 5 min and loaded into the wells of a 7.5% polyacrylamide gel along with a molecular weight marker. Electrophoresis was performed at 100 V. Silver staining was performed according to the method of Shevchenko (Shevchenko et al., Anal Chem. 1996, 68, 850-858). The main band in both samples runs at approximately 250 kDa.

# Movie legends

**Movie 1.** AFM timelapse movie (contact mode, 3-4 min/image) visualizing the formation of FN nanofibrils during membrane retraction of a MEF cells.

**Movie 2.** Imaging of vinculin-EGFP expressing MEF cells on FN-AF568 by TIRF-M. A single MEF cell expressing vinculin-EGFP spreading on FN-AF568. TIRF microscopy images were collected every minute to monitor potential FN rearrangement into fibrils. However, despite active cell spreading and focal adhesion formation, the time-lapse series demonstrate complete suppression of FN fibrillogenesis during fluorescence imaging.

**Movie 3.** AFM timelapse series of a retracting membrane area of a MEF cells. FN fibrillogenesis is inhibited after a light source is transiently switched on.

**Movie 4.** Visualizing FN fibrillogenesis in HFF cells by time-lapse fluorescence microscopy. HFF cells were seeded on FN-AF633. Images were collected every 30 min to minimize total irradiation time. Furthermore, Oxyrase® was added to the imaging medium to minimize photo damage. The imaging medium was supplemented with 1 mM Mn2+ to enhance integrin binding to FN. Scale bar 20 μm.
